# Selectins—The Two Dr. Jekyll and Mr. Hyde Faces of Adhesion Molecules—A Review

**DOI:** 10.3390/molecules25122835

**Published:** 2020-06-19

**Authors:** Igor Tvaroška, Chandrabose Selvaraj, Jaroslav Koča

**Affiliations:** 1 Central European Institute of Technology (CEITEC), Masaryk University, 62500 Brno, Czech Republic; 2 Institute of Chemistry, Slovak Academy of Sciences, 84538 Bratislava, Slovak Republic; 3 National Centre for Biomolecular Research, Faculty of Science, Masaryk University, 62500 Brno, Czech Republic

**Keywords:** selectins, selectin ligands, carbohydrate determinants, sialyl Lewis x, glycosyltransferases, inflammation, cancer, selectin-ligand interactions, inhibitors, transition state analog inhibitors

## Abstract

Selectins belong to a group of adhesion molecules that fulfill an essential role in immune and inflammatory responses and tissue healing. Selectins are glycoproteins that decode the information carried by glycan structures, and non-covalent interactions of selectins with these glycan structures mediate biological processes. The sialylated and fucosylated tetrasaccharide sLe^x^ is an essential glycan recognized by selectins. Several glycosyltransferases are responsible for the biosynthesis of the sLe^x^ tetrasaccharide. Selectins are involved in a sequence of interactions of circulated leukocytes with endothelial cells in the blood called the adhesion cascade. Recently, it has become evident that cancer cells utilize a similar adhesion cascade to promote metastases. However, like Dr. Jekyll and Mr. Hyde’s two faces, selectins also contribute to tissue destruction during some infections and inflammatory diseases. The most prominent function of selectins is associated with the initial stage of the leukocyte adhesion cascade, in which selectin binding enables tethering and rolling. The first adhesive event occurs through specific non-covalent interactions between selectins and their ligands, with glycans functioning as an interface between leukocytes or cancer cells and the endothelium. Targeting these interactions remains a principal strategy aimed at developing new therapies for the treatment of immune and inflammatory disorders and cancer. In this review, we will survey the significant contributions to and the current status of the understanding of the structure of selectins and the role of selectins in various biological processes. The potential of selectins and their ligands as therapeutic targets in chronic and acute inflammatory diseases and cancer will also be discussed. We will emphasize the structural characteristic of selectins and the catalytic mechanisms of glycosyltransferases involved in the biosynthesis of glycan recognition determinants. Furthermore, recent achievements in the synthesis of selectin inhibitors will be reviewed with a focus on the various strategies used for the development of glycosyltransferase inhibitors, including substrate analog inhibitors and transition state analog inhibitors, which are based on knowledge of the catalytic mechanism.

## 1. Introduction

The adhesion of molecules, either among cells or between an immune cell and target cellular component of the extracellular matrix, is the crucial event in the physiological process. In all organisms, these cell-cell interactions are mediated by adhesion molecules, which are highly expressed glycoproteins that mediate and regulate cell migration, survival, and apoptosis [1]. Adhesion molecules are divided into five groups: integrins, selectins, cadherins, members of the immunoglobulin superfamily, including nectins, and others such as mucins [2,3,4].

Selectins mediate cell-cell adhesion by recognizing carbohydrates presented on the cell surface [5,6]. Selectins are cell membrane glycoproteins that mediate adhesion of hematopoietic and cancer cells to endothelial cells, leukocytes, and platelets in flowing blood [7,8,9]. These adhesion events play a crucial role in inflammation, infection, cancer, lymphocyte and bone marrow stem cell homing, and immune cell surveillance. Selectins also assist homing of aberrant leukocytes in chronic and acute inflammatory diseases [10,11,12]. Recently, selectins were implicated in promoting cancer metastasis [13,14]. Selectins thus behave like Dr. Jekyll and Mr. Hyde; they have two faces, two kinds of behavior. The “good” one represents the functioning of selectins in physiological events such as inflammation, immune response, wound repair, and hemostasis. The “evil” one represents the functioning of selectins in pathophysiological processes.

Selectins belong to the group of C-type mammalian lectins that bind carbohydrate ligands in a Ca^2+^-dependent manner [9]. There are three members of the selectin subfamily: leukocyte (L)-selectin (CD62L), platelet (P)-selectin (CD62P), and endothelial (E)-selectin (CD62E). The primary sequences of the P-, L-, and E-selectins display a substantial similarity to each other and also to selectins in other species [15]. However, their structure and pattern of cell-type expression is diverse [16]. P-selectin is highly expressed in platelets, endothelial cells and stored in α-granules of platelets and Weibel-Palade bodies of endothelial cells. E-selectin is constitutively involved in leukocyte rolling and adhesion to endothelial cells and is significantly expressed in the endothelia of the bone marrow and skin. In contrast to P- and E-selectin, L-selectin is constitutively expressed on lymphocytes, monocytes, and granulocytes and is cleaved from the cell surface after cell activation. The selectins and their ligands have become therapeutic targets in the prevention or at least alleviation of various diseases, including cancer.

Several reviews on various aspects of selectins; such as selectins in inflammation and infection [10,16], selectins in cancer [13,14,17], selectins in immunity [10,18,19], the role of glycosylation in selectin interactions [19], and selectins as therapeutic targets [20,21]; have been reported over the last decade. However, whereas the role of selectin and selectin-ligand interactions in health and disease is highlighted in these reviews, the structural features of selectins and glycosyltransferases responsible for the creation of selectin ligands are lagging behind. In this comprehensive review we survey the significant contributions to and the current status of the understanding of the structure of selectins and their roles in various biological processes as well as discussing the potential of selectins and their ligands as therapeutic targets in chronic and acute inflammatory diseases and cancer. In the review, we will emphasize the structural characteristic of selectins and the catalytic mechanism of glycosyltransferases involved in biosynthesis glycan determinants. Also, the recent development in the synthesis of selectin inhibitors will be reviewed here with the focus on the various strategies used for glycosyltransferase inhibitors including transition state analog inhibitors based on the knowledge of the catalytic mechanism. Of course, due to the complexity of the subject, only selected details are discussed, but detailed coverage of this complex and multidisciplinary area of research is outside of the scope of this review.

## 2. The Structure of Selectins

Selectins are closely related cell membrane molecules composed of an *N*-terminal calcium-dependent lectin domain (CRD) responsible for carbohydrate recognition, an epidermal growth factor-like (EGF) domain, a series of consensus repeats (CR) domains, a transmembrane domain, and a short cytoplasmic tail [15]. Selectins exhibit calcium-dependent carbohydrate binding activity and are known as C-type lectins. The presence of a carbohydrate recognition domain is responsible for all three types of selectins recognizing a common motif, the sialylated and fucosylated tetrasaccharide sialyl Lewis x (sLe^x^) and its modifications displayed at the end of *O*-glycans, *N*-glycans, glycoproteins, or glycolipids [19,22]. These tetrasaccharides are the minimal glycan structures for selectins counter-receptors.

**Figure 1:**
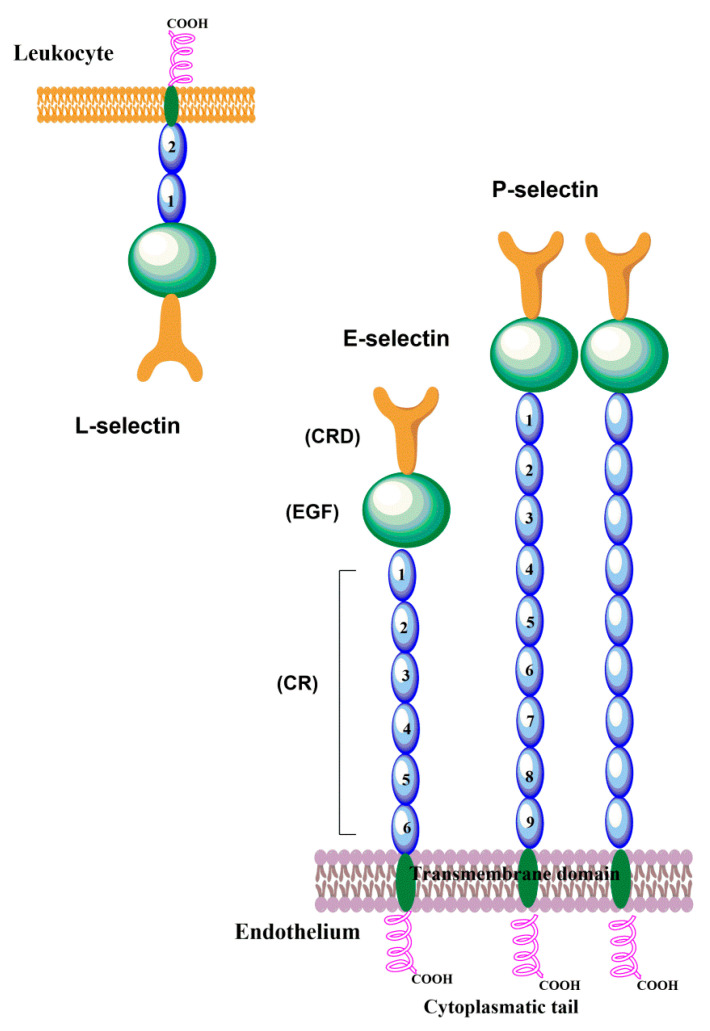
Schematic representation of P-, E-, and L- selectin structures. Selectins are closely related cell surface glycoproteins composed of five domains: the *N*-terminal calcium-dependent lectin domain responsible for carbohydrate recognition (CRD), the epidermal growth factor-like (EGF) domain, the series of consensus repeats (CR) domains, the transmembrane domain, and the short cytoplasmic tail.

Cloning and structural studies of selectins revealed their unique domain topology that regulates their function and specificity [9]. All three selectins contain the *N*-terminal lectin-like domain with 120 amino acids and with a 60–70% identity that effectively binds with carbohydrates [23,24,25,26]. The adjacent EGF-like domain also shares ~60% sequence similarity. This is followed by the CR domain with a variable number (2–9) of consensus repeats of ~60 amino acids in length, and then a short transmembrane domain and a cytoplasmic domain (Figure 1).

It was reported [24] that the sequence of human P-selectin consists of 120 amino acids, has molecular weight of about 140 kDa, and extends approximately 40 nm from the endothelial surface. While expressed on the membrane of platelets, the mass of P-selectin is about 149 kDa, under reducing conditions, and contains 28.8% carbohydrates by weight. Nine consensus repeats in a CR domain are typical for P-selectin. It associates into a homodimer by the interaction of transmembrane domains and has been suggested [27] that the dimerization of P-selectin and its ligand PSGL-1 facilitate leukocyte rolling. A small fraction of the soluble form of P-selectins (sP-selectin) is derived through mRNA splicing, which lacks the exon of the transmembrane domain. The main fraction of sP-selectin is shed into the circulation system from the activated platelets. Both the soluble and membrane form are significantly involved in the expression of stimulated endothelial cells and the platelets [24,28]. Upon activation, P-selectin is translocated within minutes onto the cell surface [15]. The maximal expression of P-selectin is 5–10 min after stimulation, and within 30–60 min P-selectin is cleared from the cell surface. This guarantees that P-selectin is only present on the endothelium surface in inflamed tissues.

E-Selectin is a glycoprotein with a molecular weight of about 116 kDa, highly expressed on the external membrane surface of vascular endothelial cells and responsible for the expression of cytokines such as tumor necrosis factor (TNF) [29]. It has a particular lectin domain, an EGF domain, and six consensus repeats of the CR domain. The amino acid sequence of the E-selectin have about six cysteine-rich consensus repeats followed by an *N*-terminal lectin domain of 119 residues, which are responsible for the binding of the oligosaccharide [9]. The EGF-like domain of E-selectin connects this domain to the stem of six consensus repeats and the bottom of the stem through single transmembrane α-helices to a small *C*-terminal cytoplasmic domain [30]. The expression of E-selectin after stimulations necessitates de novo transcription. As a result, the E-selectin is detectable on the cell surface 3–4 h after stimulation and after 16–24 h decreases back to basal levels.

L-selectin has a similar organization of extracellular domains to P- and E- selectins and has two short CRs of the complement regulatory protein domain with molecular weight of about 75 and 110 kDa depending upon the cell being investigated. The *N*-terminal nine amino acids play a crucial role in the binding mechanism between the ligand molecule and L-selectin [31]. This region is also required for the binding of lymphocytes to the high endothelial venules (HEV) of peripheral lymph nodes and the invasion of neutrophils into the site of inflammation [32,33]. L-selectin participates in the homing of leukocytes into inflamed tissues, and an increased level of L-selectin ligands initiates L-selectin–mediated adhesion events. Another critical role of L-selectin is in the adhesion of leukocytes onto leukocytes already adhered to the blood vessel wall, known as secondary tethering.

**Figure 2:**
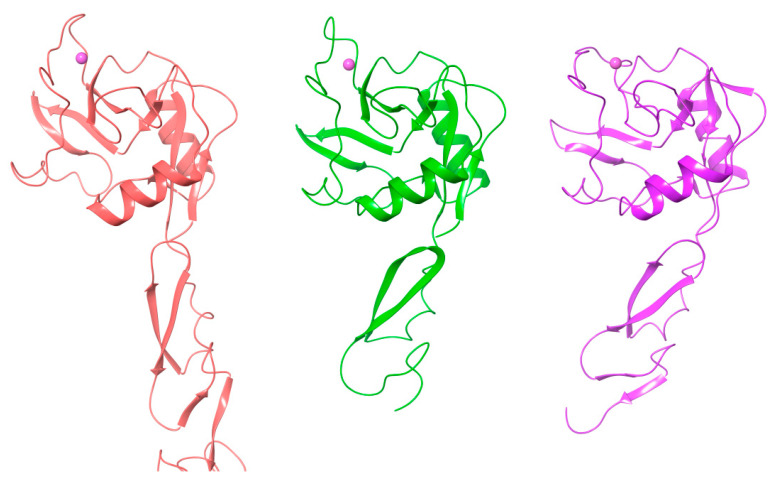
The 3-D structure of P-selectin (purple, PDB 1g1r [36]), E-selectin (red, PDB 4cs [36]), and L-selectin (green, PDB 3cfw [37]).

Although the primary structures of three lectins have been known for three decades, the 3-D structures of the selectins have been determined only recently. The structures of E-selectin [34,35] complexed with sLe^x^ and P-selectin complexed with sLe^x^ and PSGL-1 (P-selectin glycoprotein ligand) were solved [36], while for L-selectin only the structure of the lectin and EGF domains complexed with a fucose mimetic is available [37]. The crystal structures of the P-selectin construct (P-LE) containing only two domains (CRD and EGF) complexed with sLe^x^ were solved at a resolution of 2.4 Å [36]. The comparison of all three determined structures showed that all three selectins adopt a similar 3-D structure (Figure 2). The structures also revealed the same arrangement of the sLe^x^ ligand in the binding site. The critical binding interaction is electrostatic interaction between the selectin bivalent Ca^2+^ cation and the hydroxyl groups at C3 and C4 of the fucose residue of the tetrasaccharide. Further stabilization of the selectin-ligand complex is provided by hydrogen bonds between the galactose residue of sLe^x^ and Tyr4 and Glu92 of P-LE.; and between sialic acid and Tyr 48.

**Figure 3:**
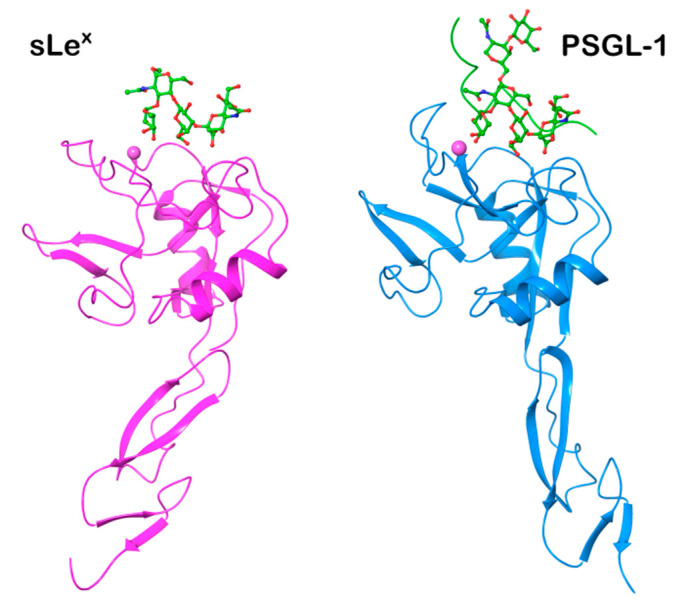
Comparison of the 3D structures of P-selectin complexed with sLe^x^ (purple, PDB 1g1r) representing the bend conformation and PSGL-1(blue, PDB 1g1s) that represents the extended, high-affinity conformation [36].

To shed some light on the different binding affinity of the selectin ligand PSGL-1 (nanomolar) compared to that of sLe^x^ (millimolar), the 3D structure of the P-LE complex with the truncated PSGL-1 construct was also determined to a resolution of 1.9 Å [36]. The crystal structures showed changes in P-LE conformation upon PSGL-1 binding. Although sLe^x^ interactions with a divalent metal cation are essential for binding, the crystal structure revealed that the interaction between PSGL-1 sulfated tyrosines and the Arg 85 and His114 of P-LE appears to be the crucial factor for the high affinity of PSGL-1. The authors suggested that P-selectin exists in two conformations: a conformation that binds sLe^x^ and an extended conformation that binds PSGL-1 with higher affinity (Figure 3). Recently, crystal structures of E-selectin complexed with glycomimetic antagonists showed [35] an extended conformation of E-selectin that represents the high-affinity binding state.

## 3. Selectin Ligands

Simple and complex carbohydrates (glycans) constitute a significant component of the outer surface of vertebrate cells (glycocalyx). They are the essential biomolecules that mediate a large number of biological and pathological events [38]. The flexibility of glycans allows them to adjust their 3-D structure to physiological needs. Thus in glycans, function determines structure, not vice versa [39]. Glycans exhibit an extraordinary heterogeneity and have a capacity to store information content that far surpasses nucleic acids and proteins [40]. This information is decoded by families of glycoproteins named lectins, to which the family of selectins belongs. In selectins, the Ca^2+^-dependent carbohydrate-recognition domains are responsible for the recognition and binding of glycans linked to glycoproteins and glycolipids. A large number of heterogeneous molecules have been shown to bind to all three selectins. However, only a few of these binders were involved in the mediation of biological processes. Criteria have been proposed [41] that characterize a real selectin ligand: (1) The ligand should be present in the right place at the right time; e.g., a true P-selectin ligand should be present on the surface of a mature circulating blood cell at a time when P-selectin is actively expressed on an endothelial or platelet surface in the vasculature; (2) The selective removal or blockade of the putative ligand on the intact cell should abrogate biologically relevant interactions; (3) The ligand should be recognized with some selectivity by the specific selectin in question, with a relatively high affinity, and preferably with well-defined stoichiometry.

### 3.1. Glycans as Minimal Recognition Determinants for Selectins

**Figure 4:**
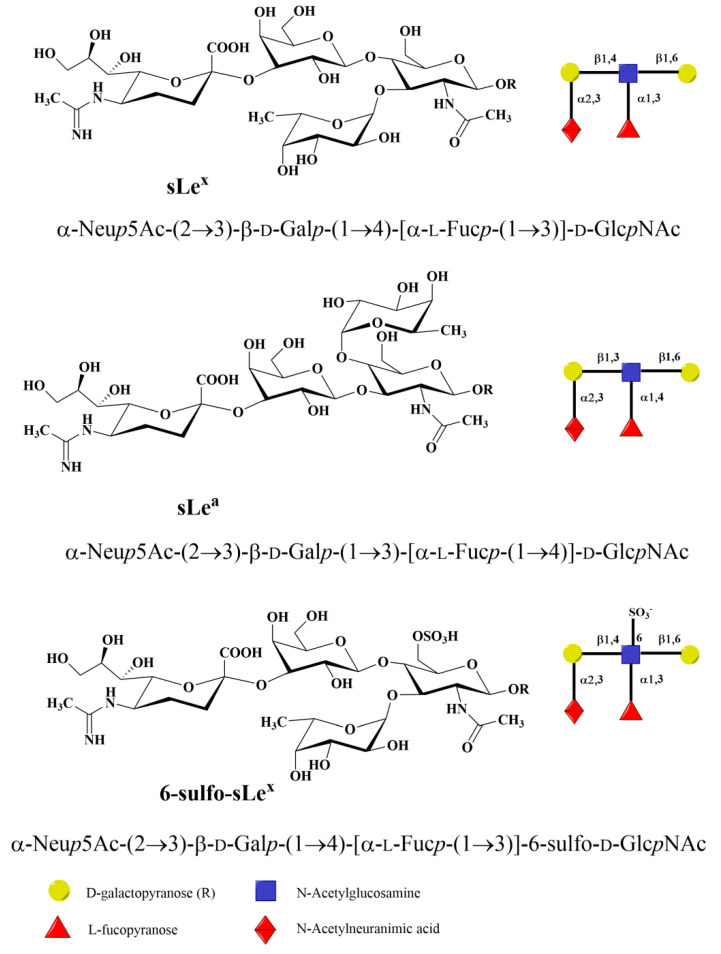
Schematic representation of minimal carbohydrate determinant structures-tetrasaccharides sLe^x^, sLe^a^, and 6-sulfo sLe^x^.

The minimal structural determinant recognized by the Ca^2+^-dependent lectin domain in all three selectins is tetrasaccharide sialyl Lewis x (sLe^x^) and its isomeric form sialyl Lewis a (sLe^a^) [22], shown in Figure 4. Selectins bind sLe^x^ with low affinity in the millimolar range. NMR experimental data [42] revealed that P-selectin exhibited millimolar affinity to sLe^x^, which is 10-fold lower than E-selectin: *K*_D_(P-selectin) = 7.8 mM, *K*_D_(E-selectin) = 0.72 mM, and *K*_D_(L-selectin) = 3.9 mM.

The molecular recognition of sLe^x^ by its respective receptors plays a crucial role in many pathophysiological events. Notably, an interaction with P-, L-, and E-selectin is of central importance in the inflammatory cascade mechanism [16]. This interaction is influenced by the conformation of the sLe^x^ that is defined by the relative orientation of the monosaccharide residues around glycosidic bonds and also by the conformation of their side chains. Molecular modeling showed that sLe^x^ and sLe^a^ are flexible molecules and in solution exist as a mixture of several conformations [43]. High-resolution NMR has been used to determine the conformation of sLe^x^ in aqueous solution and when bound to P-, E-, and L-selectin [42,44]. The analysis of the 2D transferred-NOESY spectrum indicated that P- and E-selectin bound a similar conformation of sLe^x^ while a different conformer of sLe^x^ is recognized by L-selectin. The binding studies of synthesized sLe^x^ derivatives were crucial for understanding of the structure-function relationship between the selectin molecules and carbohydrate ligands [45,46,47,48,49,50,51,52,53,54,55,56,57]. These studies not only confirmed the structure and function of the ligands but also found the interaction of essential groups of ligands to be responsible for the carbohydrate recognition mechanism and revealed that the optimal interaction of L-selectin requires GlcNAc sulfation.

The description of selectin-sLe^x^ binding modes at the atomic level was obtained by solving the crystal structures of P- and E-selectin complexed with sLe^x^ and PSGL-1. The X-ray structures of sLe^x^ bound to P- and L-selectin [35,36] revealed that interactions of the OH3 and OH4 hydroxyl groups of fucose with the Ca^2+^ ion are crucial for binding in a bent conformation. The fucose hydroxyl groups replace water molecules in free selectin structures and complete coordination of the cation Ca^2+^. In the extended binding state of E-selectin, beside the OH3 and OH4 hydroxyl group interactions with Ca^2+^ ion, additional interactions between the OH2 hydroxyl group of fucose and Glu88 of E-selectin contribute to its high-binding affinity [35].

Selectin binding determinants, carbohydrate structures that are present at the end of *O*-glycans, *N*-glycans, glycoproteins, or glycolipids, are the result of posttranslational modifications of their protein or lipid scaffolds using a repertoire of glycosyltransferases (GTs). Their biosynthesis occurs in the endoplasmic reticulum and Golgi apparatus. Glycosyltransferases are the enzymes that catalyze the transfer of a monosaccharide residue from an activated donor to the hydroxyl group of an acceptor, thus creating a new glycosidic linkage. It is assumed that there is a specific glycosyltransferase for each glycosidic linkage [58]. The donors for the catalytic reaction are nucleotide sugars, e.g., UDP-GlcNAc, UDP-GalNAc, UDP-Glc, UDP-Gal, GDP-Fuc, GDP-Man, CMP-NeuAc, etc. The side groups of synthesized glycans can be further modified; e.g., sulfotransferase modifies the hydroxyl OH6 of GlcNAc by adding a sulfate group and forms 6-sulfo-sLe^x^, which is an L-selectin ligand. The catalytic mechanism of GT was recently extensively studied both experimentally and using molecular modeling methods [59,60,61]. The biosynthesis of the selectin binding determinant tetrasaccharide sLe^x^ at the end of an *O*- and *N*-glycan will be discussed later.

### 3.2. P-Selectin Ligands

Although the binding affinity of glycan determinants sLe^x^ or sLe^a^ is in the millimolar range, they are an essential component of many glycoproteins or glycolipid ligands. These determinants are frequently present on proteins in clusters and thus involved in multivalent interactions. Multivalent interactions that dramatically enhance binding are characteristic of protein-carbohydrate interactions [62,63,64]. A large number of glycoproteins and glycolipids have been proposed to be selectin ligands, some of them with high-affinity binding. This implies that the protein backbone and its conformation contribute to selectin binding and selectivity [65].

**Figure 5:**
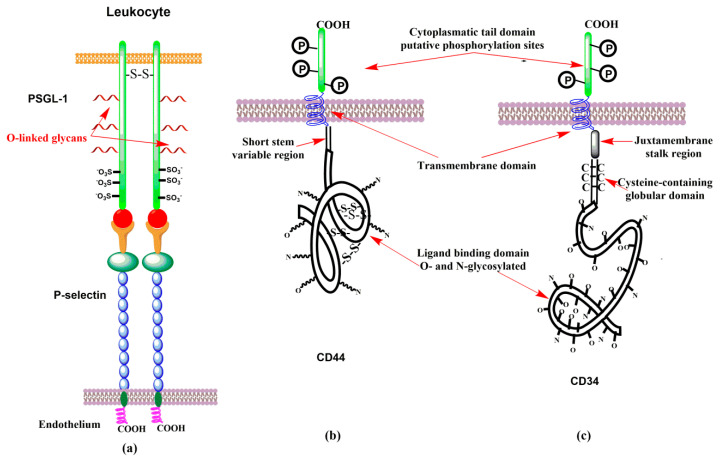
Schematic representation of (**a**) interactions of PSGL-1 homodimer with P-selectin, (**b**) CD44 glycoform, and (**c**) CD34 glycoform.

P-selectin glycoprotein ligand-1 (PSGL-1) is undoubtedly the best-described selectin ligand to date. PSGL-1 is the high-affinity ligand molecule that binds through the *N*-terminus to all three selectins but with different affinities: *K*_D_(P-selectin) = 320 nM [66], *K*_D_(E-selectin) = 15 μM, and *K*_D_(L-selectin) = 5 μM [67,68,69]. PSGL-1 is a 120 kDa disulfide-linked homodimer [70,71,72]. It was suggested that the first two biosynthesized molecules of PSGL-1 bind through non-covalent interactions, and then two cysteins near the transmembrane domain form a disulfide linkage (Figure 5a) that stabilizes the homodimer [73,74]. PSGL-1 is a transmembrane sialomucin with *O*-linked glycan determinant sLe^x^ and specific sulfated tyrosines.

PSGL-1 contains transmembrane, cytoplasmic, and extracellular domains [75]. Its transmembrane and cytoplasmic domains are highly conserved. The cytoplasmic domain binds to the Nef-associated factor and plays a crucial role in the signaling induced by P-selectin binding to PSGL-1. The extracellular domain consists of serine, threonine, and proline-rich decameric repeats that vary among species [76] and protrudes from a cell surface. The extracellular domain exhibits conformational flexibility that facilitates the binding of PSGL-1 with counter-receptors on interacting cells. The extracellular domain is *O*- and *N*-glycosylated. Despite the variability in the extracellular domain, the core *O*-glycosylation site of threonine (Thr-58) located near the N-terminus is conserved. This posttranslational modification of threonine bearing sLe^x^ permits PSGL-1 binding to selectins. Sulfated tyrosine (Tyr-46, Tyr-48, and Tyr-51) are essential for the high affinity of human PSGL-1 binding. The role of the individual sulfated tyrosines was investigated using synthesized glucosulfopeptides that modeled the binding region of PSGL-1 [68]. It was found that for binding to P-selectin, the sulfation of at least one tyrosine is important, but the sulfation of Tyr-48 (*K*_D_ ~ 6 μM) contributes to binding more than the sulfation of Tyr-46 (*K*_D_ ~ 10 μM) or Tyr-51 (*K*_D_ ~ 10 μM). A model with Tyr-46, Tyr-48, and Tyr-51 sulfated exhibited a higher binding affinity (*K*_D_ = 0.65 μM), while the non-sulfated model had considerably lower affinity (*K*_D_ ~ 25 μM). This study also suggested that the fucose contribution to binding is more significant than that of sialic acid and that all interactions are charge dependent.

The investigation of interactions between recombinant soluble P-selectin (sP-selectin) reveal that monomeric sP-selectin binds to dimeric PSGL-1 in a 2:1 stoichiometry with nanomolar affinity [55]. Interestingly, the sulfation of the GlcNAC residue of sLe^x^ that leads to 6-sulfo-sLe^x^ enhanced its L-selectin binding affinity. In contrast, the recognition of E-selectin is sulfation independent [77]. The binding of the P-, L-, and E-selectin with sLe^x^ and PSGL-1 were investigated by molecular modeling [78]. The analysis of the calculated binding free energies of selectin with ligands reveals how individual molecular moieties affect the binding affinity.

PSGL-1 is expressed on the surface of lymphoid and myeloid cells, including platelets and activated T-cells. PSGL-1 is involved through PSGL-1-P-selectin interactions in the rolling/tethering of neutrophils on endothelial cells and promotes targeted extravasation into tissues. Thus, PSGL-1 is a physiological ligand that fulfills the suggested criteria for a real ligand [41].

It was reported that CD24, also known as heat-stable antigen (HSA), binds to P-selectin. CD24 is a cell-surface glycoprotein that is highly glycosylated and was shown to mediate rolling leukocytes and cancer cells [79,80]. CD24 is expressed by neutrophils and various cancer cells and the binding of the binding of CD24 onto P-selectin is not yet clarified. It was also shown that heparin sulfate glycosaminoglycans are recognized by P-selectin [81].

### 3.3. E-Selectin Ligands

Various glycan determinants expressed on leukocytes that contain sLe^x^ and sLe^a^, and their modifications were reported to be E-selectin ligands [82]. E-selectin is the most effective at recognizing a variety of fucosylated and sialylated glycoproteins and glycolipids, while the P-selectin and L-selectin require a sulfate group on tyrosine residues and the GlcNAc residue, respectively, for a full affinity to ligands [83,84,85,86,87].

E-selectin ligand-1 (ESL-1) is a transmembrane glycoprotein that is recognized by E-selectin but not P-selectin. ESL-1 has a molecular weight of about 150 kDa and consists of a glutamine-rich N-terminal segment of 70 amino acids and cysteine-rich repeats, a transmembrane domain, and a short 13 residues cytoplasmic tail. It also contains five exclusively *N*-glycosylated sites and differs from sialomucin selectin ligands that are characterized by *O*-linked carbohydrate side-chains [88,89]. ESL-1 is expressed in many cells and primarily localized in the Golgi apparatus. A small portion of ESL-1 is also expressed on the leukocyte cell surface [90,91]. Binding studies of E-selectin with ESL-1 revealed [92] that ESL-1 requires derivatization with fucosyltransferase IV (FucT-IV) in contrast to P-selectin, which needs fucosyltransferase VII (FucT-VII). A knock-out mouse study revealed that without the presence of ESL-1, the expression of soluble E-selectin is reduced. However, when both PSGL- 1 and ESL-1 are absent the expression of E-selectin is abolished [93]. Experimental studies of the binding affinity of E-selectin to sLe^x^ estimated dissociation constants *K*_D_ between 107 μM and 1800 μM [42,48,49,94]. A significantly lower *K*_D_ = 62 μΜ was determined by SPR measurement for the dissociation of the mouse recombinant E-selectin with ESL-1 [52].

CD44 is a family of transmembrane glycoproteins (glycoforms) with a molecular weight between 80 and 220 kDa [95]. Posttranslational modifications and alternative splicing are responsible for the extreme variability in the structure and function of this adhesion molecule. CD44 is a lectin with hyaluronic acid as the primary ligand. The smallest and most abundant glycoform of CD44 is a standard one, CD44s (85–95 kDa). Human CD44s glycoprotein is composed of several domains [96] with six or seven potential sites for *N*-linked and *O*-linked glycosylation (Figure 5b), respectively. CD44s is expressed mainly on leukocytes, larger glycoforms of CD44 occur on different normal and malignant cells. Studies on L-selectin ligands led to the discovery [96,97,98] of the CD44 glycoform (90–100 kDa) that only bound to E-selectin. This glycoform was named Hematopoietic Cell E- and L-selectin Ligand (HCELL). Thus, in contrast to CD44, HCELL is the selectin ligand [99]. It was found that HCELL is expressed by human hematopoietic stem and progenitor cells [100,101], some malignancy cells [101,102,103], and by classical human monocytes [10,104]. Interestingly, human hematopoietic stem and progenitor cells exhibit the carbohydrate determinant sLe^x^ exclusively at the end of *N*-linked glycans, while classical monocytes express sLe^x^ on *O*-linked glycans of CD44s [10,104].

It has been shown [105] that the glycoform of CD44 expressed by human mesenchymal stem cells (MSC) bear α(2,3) sialylated *N*-acetyllactosamine. The selectin carbohydrate determinant is thus missing fucose α(1,3)-linked to the GlcNAc to have a complete sLe^x^ as in HCELL. To generate the HCELL selectin ligand from CD44 glycoform, the authors [105] developed so-called “Glycosyltransferase-Programmed Stereosubstitution” (GPS). They used glycosyltransferase FucT-VI for the formation of an α(1,3)-linkage between the fucose and GlcNAc to create HCELL from the native CD44 of human MSC. Further biochemical studies [106] showed that the HCELL of human MSC exhibited robust tethering and rolling interactions on marrow vessels. These results suggest that the programmed glycan engineering could be employed to increase cell delivery to tissue sites and thus in stem cell-based regenerative therapeutics.

CLA and CD43 are also E-selectin ligands. Cutaneous Lymphocyte Antigen (CLA) is a specialized glycoform of PSGL-1. CLA is a 240 kDa transmembrane sialomucin with *O*-linked glycan determinant sLe^x^ [107,108]. CLA is primarily expressed as a homodimer and is responsible for lymphocyte migration to the skin. CD43E is the 115–135 kDa glycoform of CD43 cell surface glycoprotein that displays an *O*-glycan structure with sLe^x^ at the end. CD43E is expressed on hematopoietic cells and exhibits E-selectin binding affinity [104,109].

### 3.4. L-Selectin Ligands

L-Selectin is the third member of the selectin family that binds to ligands expressed on high endothelial venules (HEV). The essential feature for L-selectin recognition is the GlcNAc sulfated sLe^x^ epitope (6-sulfo-sLe^x^) being present on biantennary *O*-glycans of numerous glycoproteins [9,110,111]. The primary physiological ligand for L-selectin is PSGL-1 though the affinity for P-selectin is considerably higher (*K*_D_(P-selectin) = 320 nM [66], versus *K*_D_(L-selectin) = 5 μM) [67,68,69].

Other L-selectin ligands have been recognized on the HEV of peripheral lymph nodes. These ligands are sialomucins belonging to the family of peripheral node addressins (PNAds), but their role in binding L-selectin is still not fully understood. A protein backbone of PNAd proteins serves as a scaffold for posttranslational modifications and the creation of proper glycan determinants. They were first detected by a monoclonal antibody, MECA-79, which identifies glycoproteins containing the carbohydrate determinant 6-sulfo sLe^x^ on core-1 *O*-glycans [110]. PNAD mucins are comprised of cell adhesion molecule-1 (MAdCAM-1), GlyCAM-1 (glycosylation-dependent cell adhesion molecules-1, Sgp50), CD34 (Sgp90), podoxalin, endomucin, nepmucin, and Spg200 [112]. L-selectin ligands are glycoproteins with various core-1 or core-2 type *O*-linked glycans capped with 6-sulfo-sLe^x^.

GlyCAM-1 is a secreted mucin-type glycoprotein from the HEV of peripheral lymph nodes, which might be responsible for a transduction of signals into leukocytes and is not involved in leukocyte adhesion [9]. In GlyCAM-1, both arms of the core-2 structure appeared to be sulfated at position C-6 of GlcNAc [113]. The binding affinity of the interaction of L-selectin with GlyCAM-1 was estimated [114] to be *K*_D_ = 108 μM. CD34 (Sp90) is expressed on the cell surface of endothelial cells. For L-selectin binding, however, CD34 is only properly glycosylated in the HEV [111,115,116]. Though the binding affinity of CD34 was not determined, it was anticipated that L-selectin binds to CD34 with similar affinity than to PSGL-1 [117]. CD34 has an extensively *O*- and *N*-glycosylated extracellular domain, a cysteine-containing globular domain, and a juxtamembrane stalk region. The single-pass transmembrane domains is followed by short cytoplasmic tails containing putative phosphorylation sites (Figure 5c) [118]. CD34 mediates the initial loose interactions of lymphocytes to HEV [119]. Another potential L-selectin ligand is mucosal addressin cell adhesion molecule-1 (MAdCAM-1). MAdCAM-1 is a transmembrane adhesion molecule with a mucin-like region that displays an L-selectin glycan determinant [120]. MAdCAM-1 supports lymphocyte tethering and rolling through interaction with L-selectin and the α4β7 integrin. Also, it has been observed that MAdCAM-1 facilitates the tethering of lymphocyte on the HEV [121,122]. Increased expression of MAdCAM-1 was observed in patient with active or chronic intestinal inflammatory diseases, which suggested a MAdCAM-1 role in lymphocyte rolling to the gut [19].

## 4. Glycosyltransferases Involved in the Biosythesis of Glycan Determinants

Glycan structures found in P-, E-, and L-selectin ligands are the result of the post-translational modification of protein or lipid scaffolds by the enzymatic process called glycosylation. The glycan structures of a ligand reflect the presence of functionalizing carbohydrate processing enzymes, such as glycoside hydrolases and glycosyltransferases. Glycosylation proceeds in a stepwise manner in the endoplasmic reticulum and Golgi apparatus. The repertoire of biosynthesized glycans is determined by the expression and specificity of the enzyme. The resulting glycans can be further modified by carbohydrate-modifying enzymes, including sulfotransferases. There are two main biosynthetic pathways of glycan synthesis that lead to *O*-linked and *N*-linked glycans. Both types of glycans may contain the terminal tetrasaccharide sLe^x^ or sLe^a^, and their modifications that are crucial for selectins binding. The subsequent action of several glycosyltransferases is responsible for the formation of these carbohydrate determinants.

Glycosyltransferases utilizing a sugar-nucleotide as the donor are called “Leloir-type” enzymes. [58,123,124]. Acceptor substrates are carbohydrates, proteins, lipids, DNA, antibiotics, or other small molecules. Glycosyltransferases exhibit low sequence homology [125]. Amino acid sequence comparisons led to their classification into 90 families, GT-1 to GT-90 [125,126]. Interestingly, despite a low homology, GTs exhibit extraordinarily conserved three-dimensional architectures. Their X-ray structures mainly show two general types of folds, termed GT-A and GT-B [60,61].

**Figure 6:**
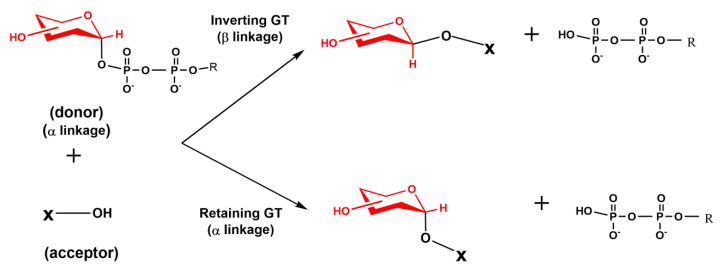
Schematic diagram of inverting and retaining reactions catalyzed by glycosyltransferases. The transferred saccharide residue is shown in red.

The chemistry of the catalytic reaction resembles a nucleophilic displacement reaction, in which the nucleophilic hydroxyl residue of an acceptor replaces the leaving group at the anomeric carbon C1 of a sugar nucleotide, e.g., a UDP moiety in the case of UDP-GlcNAc. In this glycosylation reaction, the resulting C1-O bond is oriented either axially or equatorially. Thus, based on the orientation of the glycosidic linkage, glycosyltransferases were described as either retaining or inverting enzymes (Figure 6). The structure and catalytic mechanism of GTs were recently reviewed [59,60,61]. Recent advances in characterizing the activities of glycosyltransferases and sulfotransferases using knock-out mice revealed the essential role of several GTs involved in the biosynthesis of carbohydrate determinants of selectin ligands [5,22].

### 4.1. The Glycosyltransferase Polypeptide UDP-GalNAc Transferase

**Figure 7:**
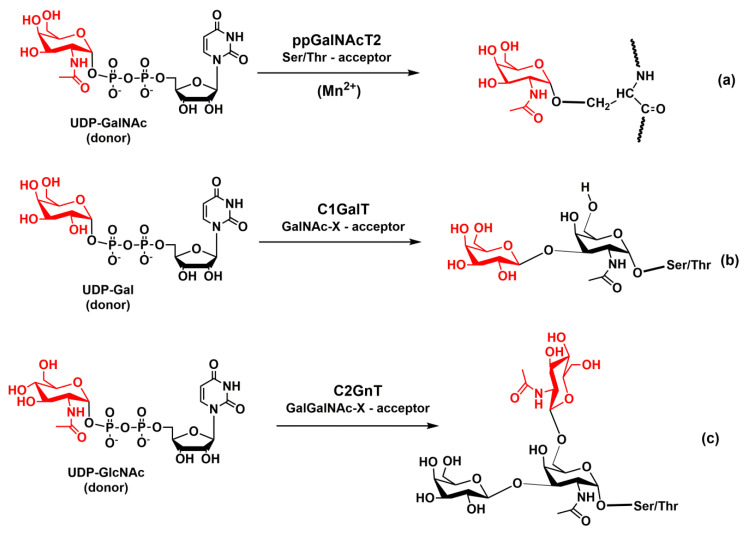
Schematic diagram of enzymatic reaction catalyzed by (**a**) ppGalNAcT2, (**b**) Core-1 GalT, and (**c**) core-2 GnT.

The biosynthesis of *O*-linked (mucin-type) glycans is initiated by replacing the hydroxyl group of the serine or threonine on a protein with an N-acetylgalactosaminyl (GalNAc) group. The reaction is catalyzed by glycosyltransferase polypeptide UDP-GalNAc transferase (ppGalNAcT2) (Figure 7a). This step is essential for the biosynthesis of carbohydrate determinants. This was demonstrated by knock-out mice lacking ppGalNAcT2 that exhibited a reduction in lymphocyte homing, rolling, and the recruitment of leukocytes into inflamed tissue [127]. Therefore, the authors suggested that the inhibition of ppGalNAcT2 activity might have therapeutic potential for some pathogenic syndromes involving increased thrombosis, chronic inflammation, and immunological diseases of B lymphocytes.

The enzyme ppGalNAcT2 exists in a large variety of isoforms [128] and the X-ray structures of human isoform 2 complexed with an acceptor and UDP [129] and isoform 10 complexed with UDP-GalNAc [130] have been solved. In both isoforms, the catalytic domain adopted a GT-A fold [131]. The ppGalNAcT2 glycosyltransferase behaves as the retaining enzyme, and its function depends on a divalent manganese ion. An investigation of the catalytic mechanism of the retaining ppGalNAcT2 transferase [132,133] showed that the catalytic reaction of ppGalNAcT2 proceeds as an S_N_i nucleophilic substitution. The calculations also determined the transition state structure of the reaction.

### 4.2. Glycosyltransferases Core-1 β-1,3-Galactosyltransferase and Core-2 β-1,6-GlcNAc-Transferase

After the addition of a GalNAc residue to a threonine or serine of a protein through α-linkage, the *O*-glycan is elongated by a core-1 β-1,3 galactosyltransferase (Core-1 GalT, C1GalT) that forms a new β-glycosidic linkage of galactose to position 3 of the GalNAc (Figure 7b). The core-1 structure is further extended by the Golgi enzyme β-1,6-GlcNAc-transferase (Core-2 GnT, C2GnT). C2GnT adds GlcNAc to the GalNAc of the core-1 structure via a β-(1,6)-linkage and forms the core-2 structure (Figure 7c). C2GnT is an inverting, metal-ion-independent enzyme. The crystal structures of murine C2GnT-L in the absence and presence of the acceptor substrate were recently resolved at a resolution of 2.0 and 2.7 Å and revealed a GT-A fold [134]. Molecular modeling supported a concerted S_N_2-like mechanism in which a nucleophilic attack by O6 and the separation of the leaving group all occur almost simultaneously [135].

The branching extension of the core-1 structure, which leads to the core-2 type *O*-glycan, is required for the biosynthesis of core-2 glycans containing the terminal tetrasaccharide sLe^x^. The C2GnT knock-out mice [136] showed that the binding of P- and L-selectin to leukocytes was almost entirely absent, and binding to E-selectin was diminished [137]. These results show that the action C2GnT-I transferase is important for the biosynthesis of P-selectin ligands, whereas for some E-selectin ligands it is not critical [138]. It was found that in the core-2 knock-out mice, the binding of P-selectin to PSGL-1 was greatly diminished, and as a result, a reduced rolling of leukocytes was observed. These results led to the conclusion that the proper functioning of P-selectin ligands require the functioning of the C2GnT-I enzyme.

In contrast, C2GnT-I only partially influences E-selectin–mediated rolling in vivo [139]. Some results suggest that there is a competition [140] between C2GnT and sialyltransferase ST6GlcNAc in the formation of the β-(1,6) linkage on the core-1 structure and as a result the biosynthesis of sLe^x^ on a core-2 glycan is altered. Since in leukocytes the expression of sLe^x^ is found exclusively on the core-2 structures, the C2GnT enzyme is a potential target for inhibiting selectin binding to granulocytes.

### 4.3. Glycosyltransferase β-1,4-Galactosyltransferase-1

**Figure 8:**
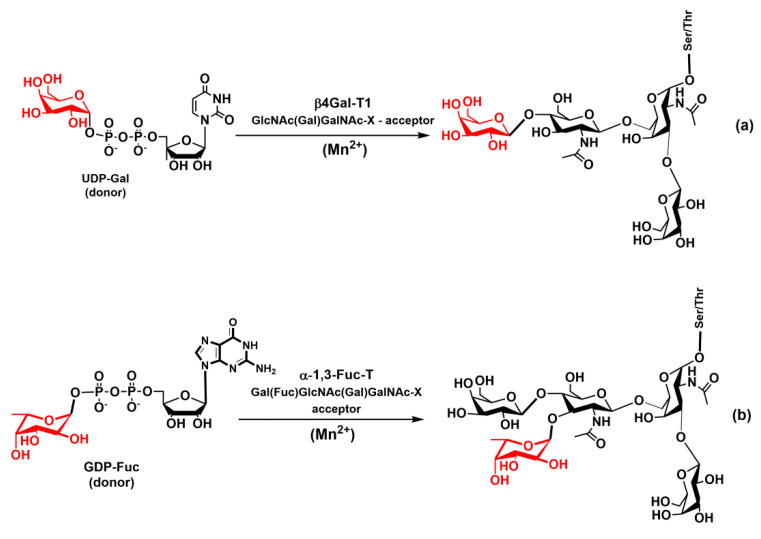
Schematic diagram of enzymatic reaction catalyzed by (**a**) β4Gal-T1 and (**b**) α1,3FucT.

The inverting β-1,4-galactosyltransferase-1 (β4Gal-T1) adds galactose to the terminal GlcNAc of the core-2 structure via a β-(1–4)-linkage, thus forming *N*-acetyllactosamine (Figure 8a). This disaccharide is crucial in the biosynthesis of selectin carbohydrate determinants. The β4Gal-T1 is the Mn^2^^+^ transition metal-dependent enzyme, and the crystal structures of the catalytic domain of β4Gal-T1 have been solved both without any substrate and complexed with substrate [141,142]. Based on the crystal structural data, an ordered sequential mechanism has been proposed for the reaction catalyzed by β4Gal-T1. A molecular modeling study supported a concerted S_N_2-type displacement mechanism [143]. How the β4Gal-T1 affects the selectin ligand activity was investigated using β4Gal-T1 knock-out mice [144,145]. The results showed significantly lower binding of P-selectin to nucleophiles, which implies a diminished recruitment of leukocytes. An analysis of *O*-glycan structures revealed a significantly reduced number of core-2 side branches and supported the role of β4Gal-T1 structures in P-selectin binding. In contrast, lymphocyte homing was not influenced in the absence of β4Gal-T1 suggesting that the biosynthesis of the L-selectin ligand was not significantly affected.

### 4.4. Glycosyltransferases α-1,3-Fucosyltransferase and α-2,3-Sialyltransferase

The biosynthesis of selectin carbohydrate epitopes continues by sequential fucosylation and sialylation of *N*-acetyllactosamine. Fucosylation is carried out by fucosyltransferases (FucTs) that catalyze the transfer of the l-fucose residue from the donor guanosine diphosphate β-l-fucose (GDP-Fuc) to various saccharides [146,147,148,149]. There are six α-1,3-fucosyltransferases (FucT-III, FucT-IV, FucT-V FucT-VI, Fuc-TVII, and FucT-IX) involved in the biosynthesis of the Le^x^ antigen [146]. Two of them, FucT-III and FucT-V, are also able to form an α-1,4-linkage to GlcNAc and thus creating the Le^a^ antigen. The glycosyltransferase α-1,3-FucT is a metal-dependent inverting enzyme with a divalent metal cation Mn^2+^ in the active site (Figure 8b). The X-ray structures of the fucosyltransferase α-1,3-Fuc-T from *Helicobacter pylori* were solved [150]. It has been proposed that the catalytic reaction of α-1,3-FucT proceeds via an S_N_1-like mechanism [150,151]. The role of α-1,3-FucT has been illustrated by FucT-VII knock-out mice that showed the abnormality of leukocyte extravasation during inflammation [152,153]. 

**Figure 9:**
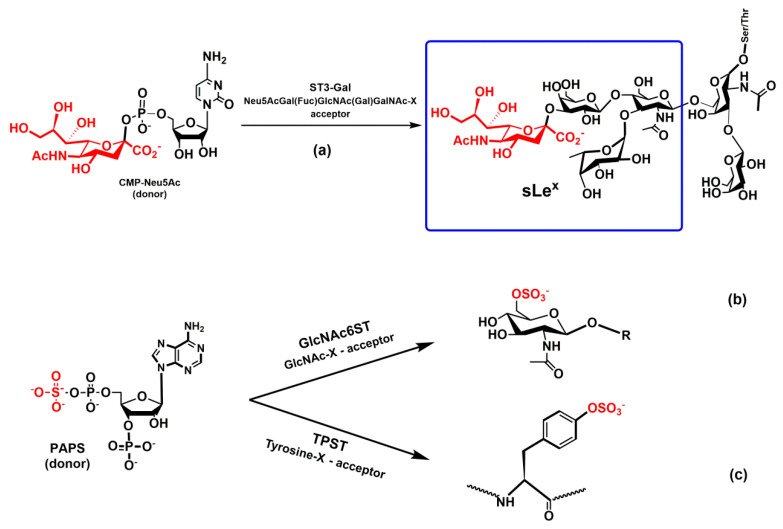
Schematic diagram of enzymatic reaction catalyzed by (**a**) ST3Gal, (**b**) GlcNAc6ST1, and (**c**) TpsT.

The biosynthesis of tetrasaccharide epitopes (sLe^x^ and sLe^a^) is completed by adding *N*-acetylneuramic acid (Neu5Ac, sialic acid) to a trisaccharide Le^x^ or Le^a^ by a sialyltransferase (ST) ST3 (Figure 9a). Sialyltransferases are grouped based on the acceptor position to which Neu5Ac is attached. This position is either α-2,3 (ST3), α-2,6 (ST6) or α-2,8 (ST8) leading to an α-glycosidic bond between the C2 atom of Neu5Ac and the 3′-, 6′-, or 8′-hydroxyl group of the acceptor, respectively [154,155]. Also, an acceptor saccharide is specified (e.g., ST3Gal-1, ST3Gal-2, etc.). STs operate as inverting glycosyltransferases, likely utilizing an S_N_2-like direct displacement mechanism [156]. The ST3Gal-1 enzyme transfers Neu5Ac from the donor cytidine-5′-monophospho-*N*-acyl-neuraminic acid (CMP-Neu5Ac) to the acceptor, which is O3 of a terminal galactose on Le^x^ or Le^a^. The crystal structures of the ST3Gal-1 and ST6Gal-1 have been solved recently [157,158,159] without the metal cation in the active site, supporting the proposal that the activity of STs does not depend on metal ions.

Knock-out ST3Gal-4 mice were generated to investigate the influence of the sialylation of a Lewis epitope on selectin ligand function [160]. The binding experiment revealed a decreased affinity of P-, E-, and L-selectin to leukocytes with knock-out ST3Gal. For example, L-selectin-dependent rolling was eliminated by removing ST3Gal-4. Interestingly, leukocytes rolling in Peyer’s patch HEV, which is mediated by unknown endothelial L-selectin ligands, was not reduced in the absence of ST3Gal-4. These results showed that PSGL-1 binding depends on ST3Gal-4, while ST3Gal-4 is not required for L-selectin ligand activity on high endothelial cells of Peyer’s patch HEV. Studies of ST3Gal knock-out mice showed an increased expression of core-2 decorated *O*-glycans with an increased binding of selectins to their ligands, suggesting that both Core-2 GnT and ST3Gal compete for the same acceptor substrate [161].

### 4.5. Sulfotransferases GlcNAc-6-Sulfotransferase and Tyrosylprotein Sulfotransferase

Sulfotransferases play an important role in the biosynthesis of selectin ligands [113,162,163,164]. An analysis of L-selectin ligand activities revealed [56] that optimal binding requires the sulfation of GlcNAc at carbon C6. Thus, an optimal ligand for L-selectin to mediate leukocyte rolling is 6-sulfo sLe^x^ and not just sLe^x^. The sulfation of GlcNAc is carried out by the enzyme GlcNAc-6-sulfotransferase (GlcNAc6ST) that transfers a sulfuryl group from the donor 3′-phosphoadenosine 5′-phosphosulfate (PAPS) to the GlcNAc of sLe^x^ (Figure 9b). The crystal structure of the Golgi resident enzyme GlcNAc6ST is not solved yet. However, a comparison of sequences of GlcNAc6ST with the known crystal structures indicated a similar structure of the catalytic site [165,166]. Recently, it has been shown that double knock-out (GlcNAc6ST-1 and GlcNAc6ST-2) mice eliminated leukocyte homing [167,168].

It is well established that the sulfation of tyrosine residues at the N-terminal of PSGL-1 increases the binding affinity of PSGL-1 to P- and L-selectin considerably [169,170]. Tyrosine sulfation is catalyzed by the Golgi enzyme called tyrosylprotein sulfotransferases (TpsT1 and TpsT2). The enzymes utilize PAPS as the donor (Figure 9c), and the structure of human TpsT2 complexed with a substrate peptide has been solved recently at a resolution of 1.9 Å [171]. Structural analysis implied an S_N_2-like mechanism that was supported by the results of molecular modeling [172]. Activity studies of PSGL-1 revealed that both isomeric forms of TpsT contribute equally to the proper function of PSGL-1 [173,174].

## 5. The Biological Role of Selectins

The adhesion of selectins to other cells or matrix components is mediated by interaction with their counter-receptors. The selectin binding interactions play a pivotal role in the many normal physiological processes and mediate the adhesion of leukocytes to endothelium, which is followed by their extravasation to the site of inflammation or injury to resolve infections and heal wounds [7,9,16,175].

Although selectins are often considered to be benign, it has been shown that selectins play a detrimental role in various acute and chronic inflammatory diseases [10,11,12]. It was observed that elevated levels of selectins often correlate with the severity of inflammatory diseases, including asthma and chronic obstructive pulmonary disease (COPD) [176,177], psoriasis [178,179,180], thrombosis [181], or arthritis [182]. The aberrant homing of leukocytes into endothelial cells mediated by P- and E-selectins plays a crucial role in atherosclerosis [183]. The role of selectins in this chronic inflammatory disease was supported by mouse experiments that showed substantially lower levels of atherosclerotic plaques in P- and E-selectin-deficient mice [184,185]. Upregulated P- and E-selectin expression observed in rheumatoid arthritis [186,187], multiple sclerosis [188], and type 1 diabetes [189] suggested their roles in the pathology of inflammatory diseases.

Moreover, selectin-ligand interactions have been associated with tumor progression and metastasis [14,17,21,190,191]. Recent experimental data support the idea that tumor cells exploit the tethering and rolling adhesion cascade employed by leukocytes to migrate through the vasculature into a particular tissue required by infection or injury [13,14,192].

### 5.1. Selectins in Inflammatory Processes

**Figure 10:**
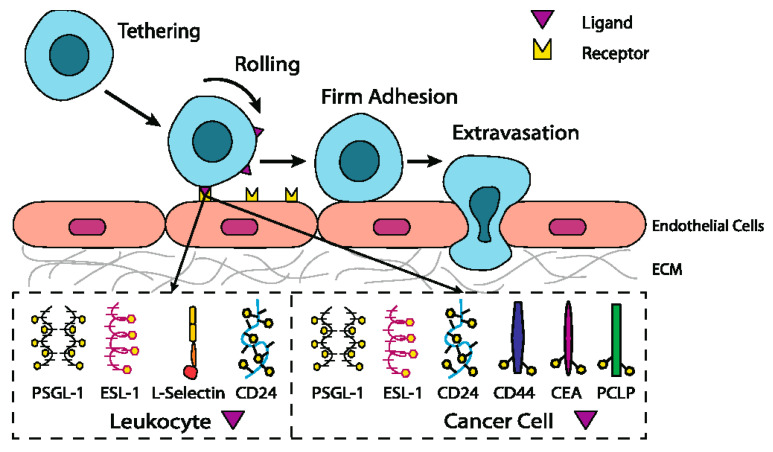
The leukocyte adhesion cascade and circulating tumor cells tethering, rolling, and firm adhesion on the endothelium share many paradigms. Most of the selectin ligands on leukocytes except L-selectin are also present on cancer cells. Recently, novel ligands on cancer cells have been observed to facilitate selectin-mediated rolling on the endothelium Reprinted with permission from [197]; copyright: Springer 2012.

Selectins are involved in leukocyte emigration from the bloodstream into tissues through multiple sequential molecular interactions (often referred to as the multi-step paradigm), including the tethering and rolling interaction of leukocytes on the surface of the activated endothelium, leukocyte activation, immobilization, and endothelial transmigration (Figure 10) [192,193,194]. It is noteworthy that all these molecular interactions occur in the bloodstream under fluid shear stress. In the first step of the inflammatory response, the circulating leukocytes in flowing blood interact with (tether to) vascular surfaces, overcome the shear forces and adhere to the endothelial surface. Then repeated rolling interactions reduce cell speeds on the surface below that of flowing blood [16,195,196]. There appears to be a correlation between the shear stress of the fluid and reversible interactions (adhesive slip and catch bonds) between rolling cells and surface [16]. Slow rolling cells decrease the distances between cells and the surface and bring them closer to another antagonist. As a result, this kind of signaling (chemokines, cytokines, etc.) activates the upregulation of integrins, which is necessary for firm adhesion and finally transmigration.

The initial interactions of tethering and rolling of leukocytes are mainly dependent on the interaction of endothelial P- and E-selectin and their principal ligand PSGL-1. The L-selectin is primarily involved during the later phase, where binding with PSGL-1 facilitates interactions between leukocytes and thus leads to an increased level of leukocytes at the inflammation site [198,199,200]. The E-selectin has been shown to act as a mediator for the adhesion cascade mechanism and was found to be responsible for an increase in the adhesion of isolated blood neutrophils in the inflammation process [201,202]. The significant role of E-selectin was described through their interaction with ligands such as ESL-1 or CD44 [203,204]. The binding of E-selectin with ESL-1 plays a key role in changing initial tethering into slower rolling, whereas the binding of CD44 affects rolling speed [93]. These interactions change the rolling of the circulating cells to stable adhesion that is also facilitated by interactions with other adhesion molecules, including integrins [205]. They also regulate selective migration; for example, naive T cells preferentially migrate into lymph nodes through the interaction of L-selectin and cells on HEV [196].

### 5.2. Mechanism of Selectin-Ligand Interaction

The tethering and rolling of leukocytes along the endothelium to the site of infection or injury requires a rapid turnover of reversible interactions between selectins and their ligands. The lectin-ligands adhesive interactions, which have been reviewed in detail [194,206,207], are specific since they occur under various shear stresses in blood flow and the motion of molecules is restricted to a two-dimensional space. The tethering and rolling also require the rapid formation (defined as the on-rate, *k*_on_) and dissociation (defined as off-rate, *k*_off_) of adhesive complexes, called “bonds”. The binding affinity constant *K*_D_ of the selectin-ligand complex is the ratio of the *k*_on_ to the *k*_off_ that characterizes the association and dissociation of the selectin-ligand bonds (complexes). Bond formation between the selectin and ligand involves many non-covalent interactions that include electrostatic interactions, hydrogen bonds, and van der Waals interactions. The strength of the non-covalent interactions between selectins and their ligands determines the extent of the binding affinity. It has been shown that a threshold shear is required by selectins for the tethering and rolling [208,209,210], and that shear of the blood flow affects the lifetime of these interactions mainly by altering their off-rates [211]. Only a few leukocytes tether and roll at shear stress below a certain threshold; when the shear stress increases, the number of tethering and rolling leukocytes increases until a maximum is reached, then any further increase in shear causes a lower number of rolling leukocytes [209,210]. Thus, the dissociation rates of selectin-ligand interactions on vascular surfaces are affected by the imposed shear forces.

The rolling behaviors of leukocytes on P-, L-, and E-selectins substrates differ as a result of the different kinetic properties of the interacting molecules. Various experiments have been performed to estimate the affinity (*K*_D_) and off-rates (*k*_off_) of selectin-ligand interactions [194,206]. Dynamic rolling assays showed that the L-selectin mediated rolling of polymorphonuclear leukocytes (PMNs) is 5–10 times faster than on P-selectin, and 8–11.5 times faster than on E-selectin substrates [208,212,213]. This was further supported by studies using knock-out mice [199,214]. Intuitively, leukocyte tethering and rolling requires rapid on- and off-rates, and based on single-molecule force spectra it was suggested that a higher *k*_off_, as well as a higher susceptibility for complex dissociation, are reasons for the faster rolling of PMNs over L-selectin compared to P- and E selectin [215]. The zero-force *k*_off_ measured for the dissociation of PSGL-1 from P-selectin was determined to be from 0.55 s^−1^ to 2.68 s^−1^ depending on the method [66,216,217,218,219]. The dissociation of PSGL-1 from L-selectin was characterized by *k*_off_ ≥ 10 s^−1^ [66,114], whereas for E-selectin, *k*_off_ = 4.6 s^−1^ was estimated [52,219]. These values are significantly higher than the values from single-molecule force spectroscopy, where 0.85 s^−1^, 0.31 s^−1^, and 0.22 s^−1^ values of *k*_off_ were estimated for L-, E-, and P-selectin interactions with PMNs [215].

Two kinetic models have been suggested for dissociation. In the slip-bond model, the lifetime of the interaction (bond) decreases, and the dissociation increases with the application of force as the result of the reduced energy barrier between the bound and free states [220]. In the alternative catch-bond model, dissociation decreases, and the lifetime of the bound state increases as a result of the deformation of the molecules that then bind more tightly [221]. The stabilization of the selectin-ligand complex by external force in the catch bond model is a unique and exciting phenomenon. The first measurements of P-selectin interactions with PSGL-1 using video microscopy [170,208,211] observed only slip bonds. The high applied forces were likely reasons for the failure of these studies to detect catch bonds. The use of atomic force microscopy, biomembrane force probes, and flow chamber experiments under a low external force led to the observation of catch-bonds [222,223,224,225]. Thus, selectin-ligand interactions follow a biphasic behavior under force, first decreasing dissociation (catch-bond) until a maximal lifetime is achieved and then increasing dissociation (slip-bond) with increasing force. Interestingly, for selectin-antibody interactions, only slip bonds were observed under force [222,223].

Based on the determined crystal structures of the P-selectin complexed with sLe^x^ and PSGL-1 [36], two major models were suggested for selectin-ligand catch bonds. Both models are based on the equilibrium of two selectin conformations, the extended high-affinity and bent conformation observed by crystallography [36]. In the allosteric model [226,227], the force alters the conformational equilibrium of two conformations of the selectin-ligand complex in favor of the extended high-affinity conformation. Thus, the force operating on the selectin-ligand complex acts as an allosteric effector and increases the ratio of the extended conformation. As the selectin extended conformation has a higher affinity to the ligand than the bent conformation, this results in an elongation of the complex lifetime and decrease in the complex dissociation represented by *k*_off_.

The sliding-rebinding model [225,228] was proposed based on molecular dynamic and Monte Carlo simulations of selectin-ligand complexes determined by crystallography [36]. The conformational behaviors of these complexes were investigated without the presence of a force. Also, the role of the force on the dissociation of selectin-ligand complexes and their conformational change was explored. The proposed sliding-rebinding model for catch bonds is the two-pathway model. In the sliding-rebinding model, various stable conformations are in equilibrium mixture, in which the stability of conformers and their transition from one conformer to another are controlled by an external force. In the absence of the force the bend conformer is most stable, while under force the extended conformer predominates. At low force, the bend conformer that predominates can dissociate along a fast pathway with a short lifetime and without much sliding and rebuilding, or slide into the extended conformer. At higher force, a conformational equilibrium is shifted towards extended conformation that helps sliding.

In the extended conformation, some interactions are broken, and new interactions are formed, or the original interactions are regenerated. This would move the complex back to the bent conformation and then to dissociation, thus slowing dissociation and extending the lifetime. The prolonged complex lifetime is characteristic for catch bonds. In the extended form, a further increase in the force decreases the transition barrier, which accelerates dissociation and the change from catch-bonds back to slip-bonds [228]. The steered molecular dynamic simulation [229] described the slip-catch model at the atomistic level. Simulations supported the sliding-rebinding mechanism and revealed that the properties of the calcium-binding site might be responsible for this behavior. There are experimental data that support both the allosteric and sliding-rebinding catch mechanism. It is likely that either model or another possible mechanism might be used by selectins depending on the structural circumstances.

### 5.3. Selectins in Hemostasis and Thrombosis

Although selectins are mainly considered to be adhesion molecules supporting leukocyte recruitment in inflammatory processes, studies over the past few decades have revealed their role in hemostasis and thrombosis [230,231,232,233]. It was found that platelet rolling on the activated endothelium, similar to the rolling of leukocytes, is mediated by the interactions of P-selectin with its ligand PSGL-1 [234]. The role of P-selectin in blood coagulation and thrombosis has been shown by in vivo experiments in baboon [235]. The investigated kinetics of thrombus formation in the arteriovenous model of thrombosis revealed that anti-P-selectins antibodies significantly block fibrin formation in the developing thrombus. P-selectin induces the activation of platelets and the adhesion of certain leukocytes to the vascular endothelial cells [236] and also upregulate tissues factor generation in monocytes. P-selectin also initiates signaling pathways in leukocytes and subsequently activates the elaboration of cytokines through a mechanism that involves the binding of P-selectin with its receptor PSGL-1 [237,238].

The binding of P-selectin with the PSGL-1 ligand also stimulates the formation of procoagulant microparticles that deliver the tissue factor to the developing thrombus [239,240]. A proteolytic cleaving of the extracellular domain of P-selectin generates a soluble form of P-selectin [241] that is secreted in plasma and also plays an important role in hemostasis [233,242]. The glycosphingolipids sulfatides that are P-selectin ligands were found to be involved in platelet aggregation [243].

The leukocytes and platelets are released together from the same organelle, and their interaction is mediated by P-selectin. An ample evidence indicates an interconnection between inflammation, coagulation, and cancer metastasis with P-selectin as a key molecule assisting this physiological interplay [11]. Taking together, the experimental data accumulated so far revealed three roles that P-selectin performs in hemostasis. First, the binding of P-selectin expressed on activated platelets and endothelial cells to their PSGL-1 ligand mediates platelet rolling. Thus, P-selectin starts a cascade of events in which platelets can more efficiently contribute to the development of the plug at the site of injury [231]. Second, P-selectin cleaved from activated platelets or endothelial cells either in a soluble form or on a microparticle interacts with PSGL-1 on the leukocyte and induce procoagulant microparticle generation containing a tissue factor [244]. Third, the interactions of P-selectin on activated platelets in thrombi with PSGL-1 on the microparticles facilitate the recruitment of procoagulant microparticles to the thrombus. The latter two functions lead to increased thrombin generation and, consequently fibrin deposition at the site of injury [231].

Several in vivo studies were carried out to explore the possible function of P-selectins in the pathogenesis of thrombosis using genetically modified animals that were deficient in P-selectins [245,246]. The results show that there were significantly lower thrombus weights in genetically modified animals, whereas wild types showed that a high circulating concentration of P-selectins caused larger thrombi. Elevated levels of sP-selectin were found in disorders associated with arterial thrombosis [242,247,248,249].

The clotting system is one of the major components that play a key role in thrombosis; the changes or abnormality of this clotting system in both anticoagulant and procoagulant systems leads to the risk of deep vein thrombosis. In this process, sP-selectin mediates the initial tethering of leukocytes and activated platelets with the endothelial cells as well as interactions with leukocytes and other platelets [181,233,243,250]. The expression of the P-selectin also influences the interrelationship between deep vein thrombosis and vein wall inflammatory response [251]. Interestingly, experimental data indicate that sP-selectin has the potential to be used as a predictive marker for future cardiovascular events [252,253]. In healthy humans, sP-selectin circulates at a concentration of about 100 ng/mL and its higher levels in the absence of injury might reflect vascular or thrombotic diseases due to its procoagulant activity [254]. For example, sP-selectin levels were found to be elevated in patients with atherosclerosis, hypertension, hyperlipidemia, myocardial infarction, and postangioplasty restenosis [255].

### 5.4. Selectins in Cancer

In the previous section, we discussed the role of P-, E-, and L-selectin in binding circulated leukocytes to the endothelium during the inflammatory response. Sufficient data supports the proposal that selectins also regulate the interactions of circulating cancer cells with endothelial cells [13,14,17,21,256,257]. Although millions of primary tumors cells intravasate into blood, only a small number of metastases develop. The process, in which cancer cells form metastases in distant organs, is not clearly understood. However, the cancer cells have to complete several consecutive steps: detachment from the primary tumor, intravasation into blood, circulation, and adhesion to endothelium, extravasation, initial seeding, and proliferation in the target tissue [258,259]. A cancer cell can only metastasize when all these phases have been completed [260]. Cancer cells interactions with leukocytes, platelets, and endothelial cells seem to be crucial for the creation of metastasis. Although cancer metastasis is not entirely understood, there is accumulating experimental evidence suggesting that the cancer cells during the metastatic cascade (Figure 10) use similar mechanisms that are employed by leukocytes in the inflammatory cascade [197,261,262,263].

Cancer cells exhibit aberrant glycosylation, and several alterations of glycan structures correlate with cancer progression [264,265,266,267,268]. The altered glycan structures vary depending on the cancer-type, but for *N*-linked glycans, they also contain antigens sLe^x^, sLe^a^, polylactosamine chains, β-(1–6)-branching, and their modifications. For mucin-type *O*-glycans, the presence of the core-1 and core-2 structures with tetrasaccharides sLe^x^ and sLe^a^ is enhanced on the cell surface. This enhanced expression is frequently associated with poor prognosis in various cancers [17,269,270]. The selectin-ligand interactions participate in the interactions of cancer cells with platelets and leukocytes, in interactions with endothelial cells, and as signal-transducing molecules [17]. Thus, selectins facilitate the spreading of tumor cells in blood and blocks the microvasculature by mediating specific interactions between the selectin-expressing host cell and the ligand on tumor cells [261].

The direct participation of P-selectins in the metastasis process was noticed with significant inhibition of the lung metastasis in P-selectin-deficient mice compared to wild-type controls [271,272]. It was suggested that P-selectin plays a decisive role in the interactions of cancer cells with platelets, thus facilitating adhesion to the endothelium and promoting metastasis. The interaction of P-selectin on activated platelets with its ligand on cancer cells leads to the formation of a platelet-rich thrombus around cancer cells that protects the cell and significantly stimulates the metastatic process [257,258,271,273,274]. In the P-selectin deficient mice, platelet-tumor cell interactions are diminished, and the thrombus was not formed [257,271,272]. Recently, flow-based cell adhesion experiments [275] showed a different influence of P- and E-selectin on the rolling adhesion of metastatic and leukocyte cells. These results revealed that metastatic but not leukocyte cells exhibit a diminished persistence of rolling adhesion in the presence of P-selectin, but no difference between cell subtypes was found with E-selectin. This finding suggests P-selectin interactions with cancer cells to be a potential target for cancer therapy.

Besides being a major adhesion receptor for leukocytes expressed on endothelial cells, E-selectin has been shown to engage in cancer metastasis [269,276,277]. The expression of E-selectin in cancer progression and metastasis has been described in several types of cancer including breast [278,279], lung [280,281], prostate cancer [282], and colorectal cancer [103].

In several animal models, E-selectin was found to enhance cancer metastasis. For example, in metastasis of the liver, an increased expression of the E-selectin was observed, whereas the down-regulation of E-selectin resulted in the reduction of metastasis [283,284]. However, in E-selectin deficient mice, the lung metastasis of colon cancer remained unaffected [285]. In contrast, the metastasis of breast cancer cells was decreased in E-selectin knock-out mice [286]. Recently, it was shown that E-selectin mediates breast cancer stem cell homing in bone marrow. This underlined the essential role of E-selectin in breast cancer metastatic relapse that can occur years after therapy [287].

In an early stage of cancer progress, cancer cells produce inflammatory cytokines that stimulate the considerable expression of E-selectin [276]. In this process, E-selectins are act as a mediator of the adhesion cascade. With disseminated cancer cells, tissue migration at distal organs is regulated through an E-selectin-dependent adhesion mechanism in which cancer cells express E-selectin ligands on their surface to make initial contact with the vascular endothelium. For example, CD44v4 is an E-selectin ligand expressed in metastatic breast cancer. CD44v4 regulates the interaction of the cancer cell with endothelial cells via E-selectin to facilitate the transendothelial migration of the cancer cell [284,288].

Various cancer cells express molecules that interact with L-selectin and may, therefore, mediate metastasis. Experiments with L-selectin-deficient mice showed a decrease in metastasis and confirmed the role of L-selectin in metastasis [289,290]. Moreover, the synergetic effect on a decrease in metastasis by P- and L-selectin double knock-out mice was observed [291], thus further supporting the active role of both selectins in metastasis. In the tumor microenvironment, key components are inflammatory cells that produce pro-inflammatory cytokines that up-regulate selection expression. The recruitment of leukocytes by L-selectins to activated endothelial cells may be potential way to enhance metastasis. The tumor microenvironment is affected by the presence of inflammatory cells, and their increased level usually correlates with primary tumor growth as well as metastasis [292,293]. It was reported that the formation of a permissive microenvironment and pre-metastatic niche are crucial for the establishment of metastases and are responsible for circulating cancer cells being able to colonize distant organs. Selectins and their respective ligands also contribute to maintaining the shape of the pre-metastatic niche [294,295,296].

Selectins perform a dual role in cancer. While selectins mediate metastasis by using the inflammatory cascade and participating in the shape of the tumor microenvironment, they also contribute to recognizing and killing tumor cells [3,18,191]. To target cancer cells, the immune system generates T cell responses that identify and eliminate cellular alterations that are characteristic for a given cancer. The effective destroying of cancer cells proceeds in several steps, known as the cancer-immunity cycle, and selectins are indicated in the infiltration of T cells into tumor sites [191].

### 5.5. Signaling Functions of Selectins and Selectin Ligands

In addition to their role as initiators of the adhesion events in physiological and pathological processes, selectin and selectin ligands also operate as signal transducers [16,261,297,298]. The signaling mediated by selectins was first supported by myeloid cell adhesion to P-selectin on an endothelial cell in vitro [238,272,299,300]. Several studies imply that selectins trigger signals that modulate β2 integrin functions and mediate slow rolling of neutrophils [301]. The interaction of P-selectins with PSGL-1 and E-selectins with PSGL-1 or CD44 trigger the neutrophil signaling cascade that involves the activation of tyrosine kinases and recruitment of an adaptor leading to a change in integrin conformation [302]. The interaction of platelets with PSGL-1 on myeloid cells initiates the signaling through the selectin molecule [303]. Interacting platelets and myeloid cells are in contact with various mediators, and platelets express some chemokines that activate integrins and induce the expression of other mediators in myeloid cells [238]. In vitro experiments helped to understand the roles of selectin in signal transduction that influence cell migration as well as the activation of other adhesion molecules including integrins. However, they are not yet fully understood. Specifically, selectin-mediated signaling in vivo, where other mediators are present, remains to be clarified. 

## 6. The inhibition of Selectin-Ligand Interactions

Numerous studies, including both in vitro and in vivo models, have demonstrated the essential role of selectins in various physiological processes and also reported the significant body of information on numerous inflammatory diseases and cancer. As a result, the selectins and selectin ligands represent a promising therapeutic target for the treatment of diseases, such as acute or chronic inflammatory diseases or metastatic cancer. Different types of strategies have been used to inhibit selection functions. The approaches include the modulation of selectin-ligand interaction, selectin expression, alteration of the biosynthesis of selectin ligands, and cleaving the selectin ligand [11,20,256,304,305].

### 6.1. Inhibition of the Expression of Selectins

**Figure 11:**
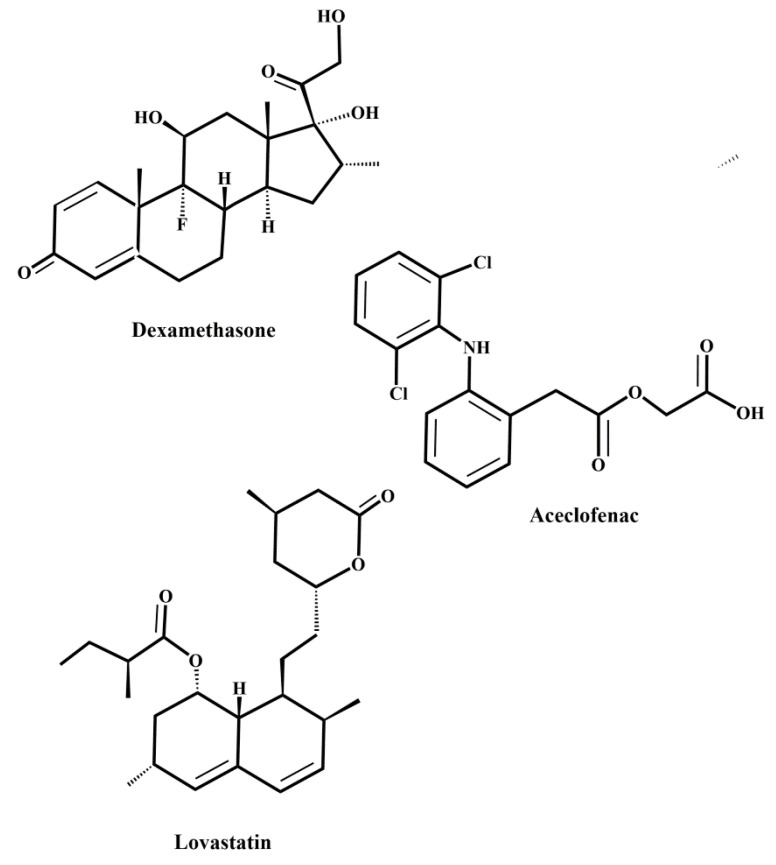
Schematic representations of dexamethasone, lovastatin, and aceclofenac, which are agents involved in the inhibition of selectin expression.

Selectin’s functions can be disrupted by inhibiting their expression. This approach was used to alter the progression of inflammation processes. Several compounds were patented for the treatment of inflammatory diseases and cancer by inhibiting the expression of selectins via various pathways and with differing success. Here we mention three agents that are widely used for the treatment of inflammation and cancer, namely dexamethasone, aceclofenac, and lovastatin (Figure 11). It is claimed that inhibition of the expression of selectins contribute to the mechanism of action of these agents.

Dexamethasone is a synthetic corticosteroid and is used to treat many inflammatory, autoimmune conditions and cancer. Dexamethasone was developed by Merck in 1957 [306] and was approved for medical use in 1961. The molecular mechanism of dexamethasone’s action was investigated in porcine aortic endothelial cells [307]. The authors observed that in the presence of dexamethasone, the E-selectin expression induced by proinflammatory stimuli is reduced markedly and identified nuclear factor-kappaB as the primary target for corticosteroid-mediated E-selectin repression.

Aceclofenac is a nonsteroidal anti-inflammatory drug (NSAID) that has a fast and potent effect on the expression of cell adhesion molecules. Aceclofenac was developed in 1991 to improve the gastrointestinal tolerability of diclofenac [308] and is used for the treatment of chronic inflammatory conditions such as osteoarthritis, rheumatoid arthritis, and in pain management [309,310]. In vitro adhesion assays using flow cytometry showed that aceclofenac caused a dramatic decrease in L-selectin expression and significantly reduced the L-selectin dependent neutrophil adhesion to endothelial cells [311].

Lovastatin belongs to a group of drugs known as statins that are inhibitors of 3-hydroxy-3-methylglutaryl-coenzyme A reductase. Lovastatin was discovered in the late 1970s at Merck Research Laboratories (Kenilworth, NJ, USA) and is a natural compound obtainable, for example, from red rice [312] or Pu-erh tea [313]. Lovastatin is a prodrug and is widely used as a cholesterol-lowering drug and for the treatment of cardiovascular disease. Several studies have suggested anti-tumor and immunomodulatory properties of lovastatin [314,315]. It was found that lovastatin decreases the expression of E-selectin [316]. It was also shown that lovastatin reduces inflammatory and fibrotic response in a mouse model [317].

### 6.2. Glycomimetic Inhibitors

The inhibition of selectin-ligand interactions as a tool for altering selectin activity is, without a doubt, the most commonly employed approach. Different types of strategies were used to design selectin antagonists, including carbohydrates, glycomimetics, non-carbohydrate small molecules, macromolecules, peptides, monoclonal antibodies, and DNA-aptamers. These efforts were reviewed recently [11,20,256,304,305,318,319,320,321,322,323,324].

**Figure 12:**
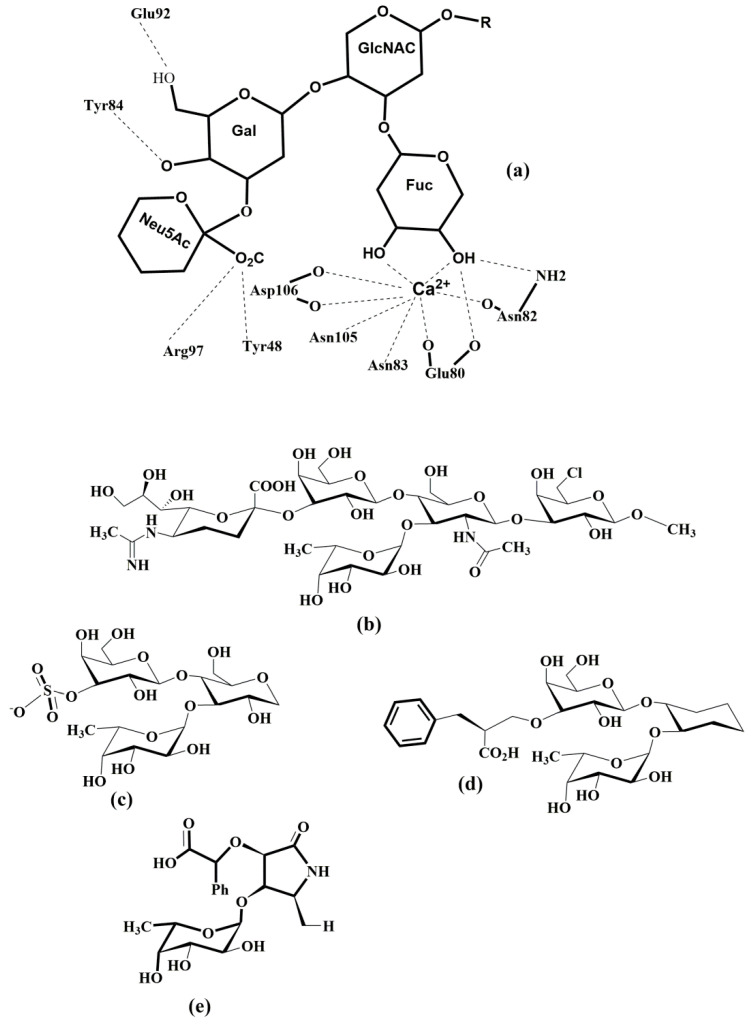
Schematic representation of (**a**) binding interactions of sLe^x^ with E-selectin, (**b**) the selectin antagonist Cylexin [326], and selected glycomimetics containing (**c**) three sugars [327], (**d**) two sugars [328], and (**e**) one sugar [329].

The tetrasaccharides sLe^x^, sLe^a^, and 6-sulfo-sLe^x^ have been identified as the minimal structural determinants recognized by CRD of all three selectins. The design of carbohydrate analogs of sLe^x^ with improved potency appeared to be challenging. Indeed, the synthesized analog of sLe^x^, pentasaccharide celexin (CY-1053) (Figure 12b), though useful in blocking selectins, has pharmacokinetics parameters that are unsuitable for a drug candidate [321]. The likely reasons were the low metabolic stability and the rapid clearance of carbohydrates. An additional reason may be the weak binding interactions of sugars with proteins, including selectins. The *K*_D_ values are usually in the millimolar range, and as a result, native ligands are quickly replaced by bulk solvent, influencing residence time. Glycomimetics, chemically modified analogs of carbohydrates, have been used to overcome these problems. The main goal of glycomimetics is to improve drug-like properties, increase selectivity and potency, and enhance stability compared to native sLe^x^. Numerous studies focused on the development of orally-administrated, potent, low molecular weight selectin antagonists.

Various strategies were used, including the replacement of an endo- or exo-oxygen atom with another atom, the replacement of functional groups, replacement of a glycosidic bond with a non-glycosidic bond, replacement of a sugar ring with an acyclic linker, utilizing the region near the binding site to increase potency, or improving the binding affinity by using a multivalency concept. Sialic acid bearing an anionic carboxylate group is another complicating factor in designing glycomimetics, and was often replaced with sulfate or phosphate [20,318,319,325]. The rational design of a glycomimetic requires an understanding of the interactions between the carbohydrate determinant (sLe^x^, sLe^a^**,** and 6-sulfo-sLe^x^) and selectin.

Experimental and molecular modeling methods can be used as tools to identify essential interactions responsible for the specificity and potency of natural ligands. The recently determined crystal structures of sLe^x^-selectin complexes [34,35,36] together with the NMR determined [42,44,57] bound conformation of sLe^x^ provided this information (Figure 12a). Many medicinal studies focused on the design of selectin antagonists using bioactive conformation of sLe^x^ as a guide. Numerous glycomimetics were synthesized containing one, two or three monosaccharides residues (see examples in Figure 12), the most potent small-molecule inhibitors having activities in the low micromolar range [20,304,319,327,330,331,332,333,334,335,336,337,338,339,340,341,342].

Studies of sLe^x^ analogs showed that crucial interactions for binding, from an inhibitor standpoint, are provided by fucose and galactose residues of sLe^x^, while the primary function of the GlcNAc residue seems to be properly orienting the fucose and galactose residue (Figure 12a). Similarly, it was shown that sialic acid had little effect on the binding to E-selectin [48,343]. Thus, it was suggested to replace the GlcNAc residue with an acyclic tether with defined conformational bias [344], which led to the design and synthesis of selectin antagonists that exhibited the required activity both in vitro and in vivo [345]. Recently, several E-selectin inhibitors that have potential for anti-inflammatory drug development were designed with an approach that targets neighboring regions of the binding site [346]. The authors used a fragment-based screening using NMR to identify fragments that interact with sites surrounding the CRD of E-selectin and prepared several compounds by connecting an sLe^x^ mimetic via a triazole linker to the second-site ligand exhibiting nM binding affinity. 

Carbohydrate-lectin interactions are characterized as multivalent interactions [64]. Different types of multivalent glycoconjugates bearing sLe^x^ or their analogs were prepared to mimic the multivalent arrangement of natural selectin ligands. They exhibited a considerably higher inhibition of selectins than their monomeric derivatives [347,348,349]. Numerous synthetic approaches were designed to produce glyco-clusters [64] with promising results, exhibiting nanomolar inhibitory activity against P- and L-selectin [350,351,352]. Unfortunately, despite a high potency, these multivalent compounds do not have drug-like properties and its remains to be seen whether they can be developed as oral drugs.

### 6.3. Macromolecular Inhibitors

**Figure 13:**
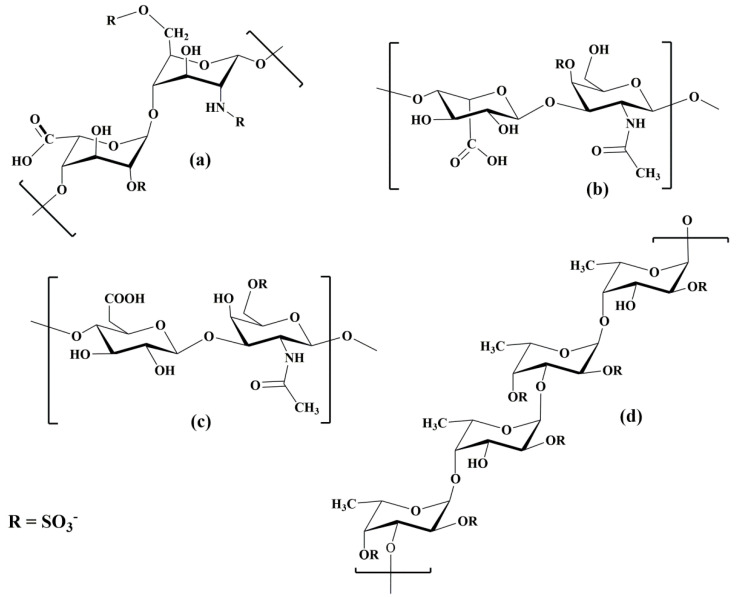
Schematic representation of natural sulfated polysaccharides with inhibitory activity against selectins; (**a**) heparin, (**b**) dermatan sulfate, (**c**) chondroitin sulfate, and (**d**) fucoidan.

Another group of selectin inhibitors of carbohydrate origin consists of negatively charged polysaccharides, and a wide range of sulfated polysaccharides (Figure 13) have been described to modulate the activity of selectins. Heparin and its derivatives [353] that have been used for years as anticoagulants have been shown to intervene with selectin-ligand interactions exhibiting anti-inflammatory and anti-metastatic activity [257,354]. Experiments with P- and L-selectin mice indicated that heparin attenuates metastasis by inhibiting P- and L-selectin [272]. 

It is noteworthy that heparin does not seem to influence E-selectin [355]. Also, the effect of heparin in various inflammatory conditions has been investigated in clinical trials with different results [11]. Other poly-anionic polysaccharides have also been investigated as selectin inhibitors. These include dermatan sulfate, which exhibits inhibitory activity against P- and L-selectin [356]. A similar activity has been observed for chondroitin sulfate [357] and sulfated chitosan [358].

Recently, fucoidan, a natural sulfated polysaccharide with a backbone structure formed of sulfated α-L-fucose, gained attention for its selectin binding properties [359,360,361]. The anti-cancer properties of fucoidan were investigated for a variety of cancers [360,362]. It has been shown that P- and L-selectin inhibition by fucoidan interferes with tumor cell-platelet interaction [363]. Surface plasmon resonance was used to estimate the binding properties of low-molecular-weight fucoidan, heparin, and dextran sulfate. The results revealed that the inhibitory activity *IC*_50_ of fucoidan was 20 nM compared to 400 nM for heparin and <25 000 nM for dextran sulfate [364]. The authors also found that heparin and dextran sulfate exhibit dissociation constant two orders of magnitude greater than the *K*_D_ = 1.2 nM for fucoidan. Though fucoidan has promising therapeutic potential in cancer treatment, its structural variety and limited knowledge about its properties will require further studies [359].

Monoclonal antibodies (mAbs) that bind to a selectin determinant were used in many experiments studying inflammatory diseases by recognizing surface-expressed P-selectin [365] and were the first meaningful P-selectin inhibitors. However, the pharmacokinetic properties of mAbs and their specificity limit their use as therapeutics. For example, the E-selectin monoclonal antibody CDP 850 developed by CELLTECH for treating psoriasis, though well-tolerated, did not possess a therapeutic effect [366] and its Phase-II clinical trial was discontinued.

Recently, an application of nanoparticles for drug delivery has been extensively investigated [367,368], and the specific expression of P- and E-selectins on the surface of endothelial cells make them outstanding targets for the nanoscale targeted delivery of drugs. Therefore, P- and E-selectin were explored for the site-selective delivery of therapeutic agents to an activated endothelium, and some studies have showed the efficacy of targeting P- and E-selectin in inflammation and cancer. Various liposomes conjugated with mAb H18/7 that recognize E-selectin were used to deliver both fluorescent probes and toxic compounds to activated HUVECs [369]. The results indicated the potential of this approach in the treatment of various cardiovascular diseases. In another study, it was observed that polyethylene glycol (PEG)-derived lipids conjugated with mAbs were able to reach and bind E-selectin in vitro, both in static and dynamic conditions and suggested that they can be used as a drug delivery system [370]. Polymeric microspheres functionalized with different carbohydrate ligands for selectins were used to mimic P- and E-selectin-mediated rolling [367] and have potential for targeted drug delivery. Different chemotherapeutic drugs, including paclitaxel and doxorubicin, were encapsulated in a nanoparticle based on the polysaccharide fucoidan to target tumors via P-selectin interactions [368]. The nanoparticles improved drug delivery in both primary and metastatic models and exhibit extraordinary efficacy. 

### 6.4. Non-Carbohydrate Inhibitors

**Figure 14:**
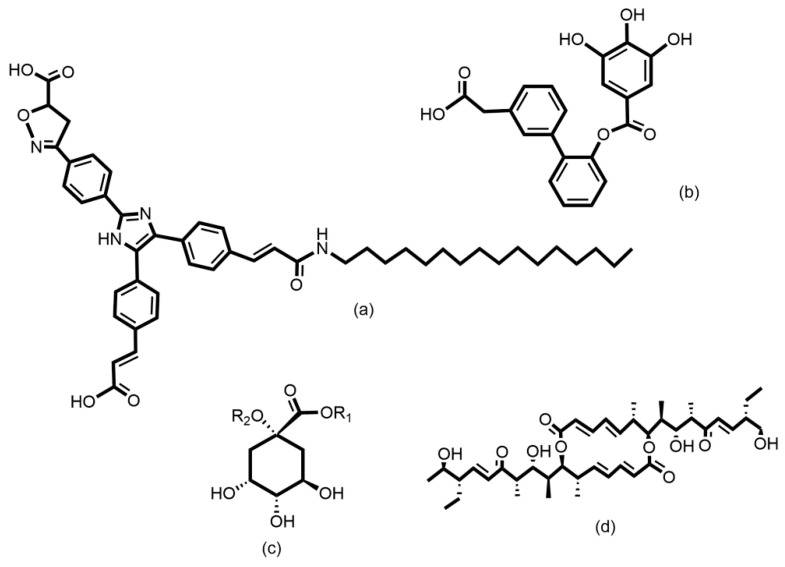
Schematic representation of selectin non-carbohydrate inhibitors: (**a**) imidazole-based derivative [371] (*IC*_50_ = 290 nM against P-selectin), (**b**) gallic acid analog [372] (*IC*_50_ = 870 nM against P-selectin), (**c**) quinic acid derivative [373], and (**d**) efomycin M [374].

Although carbohydrate-based inhibitors mimic the essential binding motifs of sLe^x^, their pharmacokinetic properties are usually not suitable for drug development, because their potency is only moderate and they are often challenging to synthesize. As a result, there has been considerable effort to utilize different scaffolds, and non-carbohydrate inhibitors represent an attractive alternative exhibiting better pharmacokinetic profile. The high throughput screening of a novel series of synthesized imidazole-based compounds was used to discover the potency of non-carbohydrate small molecule inhibitors with anti-inflammatory activity [371]. This procedure led to an inhibitor with significantly enhanced potency, from *IC*_50_ = 17 μM for an original lead to *IC*_50_ = 290 nM for the new lead compound. The structure activity relationship (SAR) of these inhibitors revealed three essential criteria [371] for potency: (1) calcium-binding moiety, (2) a rigid template/core, and (3) a hydrophobic moiety. One of the more potent compounds (Figure 14a) exhibited activity in all in vitro assays and reduced inflammation in vivo and also inhibited selectin-mediated rolling.

Various quinic acid derivatives (Figure 14c) were designed as non-carbohydrate selectin antagonists using a combination of X-ray and molecular modeling methods [373]. Structure-based design led to compounds with *IC*_50_ = 100–1000 μM in the rolling assay. A combination of synthesis and structure-based design was used in another study to develop a potent non-carbohydrate pan-selectin inhibitor [375]. The authors used a pharmacophore model based on molecular dynamics simulations of selectin- ligand complexes [376] that contains three features: (1) one of the carboxylic acids coordinates calcium; (2) the other carboxylic acid is located at a distance of 8–9 Å and forms electrostatic interactions with the Lys or Arg in selectin; (3) a long alkyl chain interacts with the hydrophobic region of selectin. The best compounds inhibited the rolling and adhesion of leukocytes with *IC*_50_ = 28 μM. In another study, a ligand-based approach was used to discover novel small molecule non-carbohydrate and non-peptidic pan-selectin inhibitor bearing a trihydroxybenzene moiety (Figure 14b) with a molecular weight below 500 [372]. A small-molecule antagonist of P- and E-selectin efomycin M (Figure 14d) was chemically prepared from fermentation material of *Streptomyces BS1261* [179]. The authors showed that efomycin M effectively reduced selectin-mediated leukocyte adhesion and exhibited therapeutic efficacy in mouse models of psoriasis. The combination of NMR methods together with molecular modeling revealed a similarity of the three-dimensional structures of efomycin M and sLe^x^, and, therefore, it was suggested that efomycin M is a selectin antagonist. This mode of action is still unclear, since one study questioned this mechanism [377] while another study supported it [378]. Later, the total synthesis of efomycin M was published [379].

Recently, several attempts to design peptide inhibitors that modulate selectin-PSGL-1 interactions have been carried out [20,304,321]. Synthetic sulfopeptides were designed to mimic the N-terminal domain of PSGL-1 [67,68]. Phage-display libraries were used to discover small peptides that bind specifically to selectins [380,381,382]. The identified heptapeptide IELLQAR was found to inhibit selectin binding to monocytes and subsequently attenuated atherosclerosis. It has been shown that this peptide analog of a selectin ligand inhibits the binding of P-selectin to monocytes with *IC*_50_ = 5 μM [380,382]. Another group discovered a new class of small P-selectin inhibitors based on an EWVDV moiety. This peptide inhibits the P-selectin–PSGL-1 interaction in a specific and calcium-dependent matter and exhibited an affinity of *IC*_50_ = 12 μM and *IC*_50_ = 8 μM for human P-selectin in an HL60 and ELISA adhesion assay, respectively. Interestingly, the potency of the peptide was increased 200-fold when present in the tetrameric form [381]. Their specificity, high potency, and affordable synthesis make these peptides mimicking PSGL-1 promising candidates for new therapeutics.

### 6.5. Compounds in Clinical Trials

**Figure 15:**
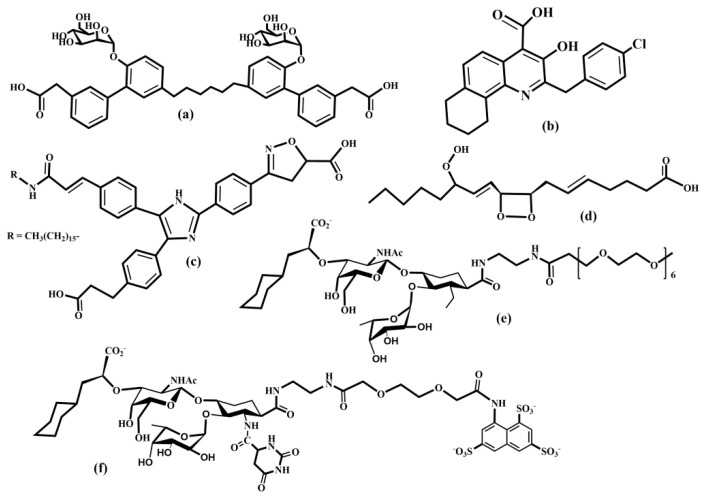
Selectin inhibitors in preclinical and clinical trials: (**a**) Bimosiamose, (**b**) PSI-697, (**c**) OC-229648, (**d**) PGX200, (**e**) uproleselan, and (**f**) rivipansel.

Over the last three decades, numbers of small-molecule inhibitors of selectins have been reported with varying potency and many of them have been validated in animal models. However, despite this enormous effort, only a few compounds have entered clinical trials, usually without success [20,176,304,320,324]. Some examples of recently developed small-molecule selectin inhibitors are given in Figure 15.

Bimosiamose is a synthetic compound [355] that inhibits all three selectins and is one of the leading selectin inhibitors currently in clinical development. Bimosiamose is pan-selectin inhibitor being developed by Revotar Biopharmaceuticals (Hennigsdorf, Brandenburg, Germany) as an anti-inflammatory drug for the treatment of acute and chronic inflammatory and immunological disorders. In vitro assays using glycolipids containing sLe^x^ showed that Bimosiamose is a competitive inhibitor of selectin binding sLe^x^. This compound has shown encouraging results in a phase IIa trial in patients with asthma, psoriasis, and COPD. After intravenous administration, patients showed a weakening of airway inflammation and lung function improvements were observed. These findings support the potential of Bimosiamose as a new anti-inflammatory therapy for COPD [383].

Uproleselan is a specific inhibitor of E-selectin developed by GlycoMimetics (Rockville, MD, USA) for the treatment of acute myeloid leukemia (AML) and potentially other hematologic cancers. Uproleselan is a synthetic glycomimetic and has been shown to disrupt cell survival pathway activation, enhance chemotherapy response, and protect from toxicity with improved survival in vivo. A phase I/II study of uproleselan added to chemotherapy in AML showed promising remission rates and survival outcomes [384]. A pivotal phase III study is underway to assess the efficacy and safety of uproleselan with standard salvage chemotherapy [385]. It is noteworthy that GlycoMimetics scientists have rationally designed an innovative antagonist of E-selectin, GMI-1687, that is suitable for subcutaneous administration. GMI-1687 has been observed to have an equivalent activity to uproleselan in preclinical models but at an approximately 250-fold lower dose.

A less successful story is that of rivipansel (GMI-1070). Rivipansel is a synthetic, glycomimetic molecule and pan-selectin antagonist, and a potential treatment for the vasoocclusive crisis associated with sickle cell anemia. It was developed by GlycoMimetics in partnership with Pfizer (New York, NY, USA). By preventing selectin-mediated cell adhesion in sickle-cell anemia, this agent may inhibit red blood cell-white blood cell interactions, normalize blood flow, and reduce inflammation and vascular occlusive pain. The results for rivipansel Phase I and II clinical trials supported the progression of the potential treatment to Phase III clinical trials [386], but failed to meet its treatment goals. Several other inhibitors are in preclinical or Phase I trials, for example, PGX-200, PSI-697, OC-229–648 (Figure 15), quinic acid derivatives, and efomycin M (Figure 14).

## 7. Carbohydrate Processing Inhibitors

In the development of selectin inhibitors, a great deal of effort has focussed on seeking mimetics of sLe^x^ involved in selectin-ligand interactions, as discussed above. These molecules, so-called blockers, are expected to occupy carbohydrate-binding sites on selectins and thus block the interaction. Another strategy for interfering with selectin-ligand interactions is the development of molecules that alter the biosynthesis of carbohydrate determinant structures, so-called carbohydrate processing inhibitors (CPIs). Carbohydrate processing inhibitors block the recognition function of selectins by preventing the specific carbohydrate determinants from being synthesized. Thus, CPIs are, in principle, much more effective than “blockers” because the ligands are so structurally altered that they cannot be recognized at all.

However, the development of CPIs has shown to be challenging due to several factors. Glycosylation occurs in the endoplasmic reticulum and the Golgi apparatus, which means that inhibitors must be able to access the correct cellular compartment where the targeted GT operates. The inhibitor must exceed the affinity of the natural substrate, which is usually in the millimolar-micromolar range. Since several GTs utilize the same donor substrates and show a certain homology of the active site, the inhibitor must also demonstrate high specificity for the targeted GT. The catalytic mechanism of GTs is complex and requires that the natural substrates, i.e., donor and acceptor, meet in the catalytic site for the catalytic reaction to proceed. Thus, CPIs can be divided into three groups: substrate analog inhibitors, transition-state analog inhibitors, and non-carbohydrate inhibitors. Several excellent reviews have been recently published that document progress in this area [387,388,389,390,391,392,393,394,395]. It is noteworthy that despite their enormous therapeutic potential, only two GT inhibitors are in clinical use. Unfortunately, neither of them inhibit the GT involved in the formation of the carbohydrate determinant. The iminosugar *N*-butyl-deoxynojiromicin (miglustat, Zavesca) [396], used in the treatment of Gaucher disease, inhibits glucosylceramide synthase, and ethambutol [397] is an inhibitor of arabinosyltransferases (tuberculosis). This probably reflects the main drawback of the majority of GT inhibitors, that they are donor or acceptor analogs with poor pharmacokinetic properties.

### 7.1. Substrate Analog Inhibitors

Glycosyltransferases catalyze the transfer of a monosaccharide residue from a sugar-nucleotide donor to the hydroxyl group of an acceptor. Therefore, it is natural that the development of substrate analog inhibitors for GT has used three different approaches: (1) sugar-nucleotide donor analogs, (2) acceptor analogs, and (3) bi-substrate analogs, in which the donor and acceptor analogs are covalently bound. Initially, these analogs were developed for mechanistic and structural studies of recombinant GTs. The drawback of these approaches is that substrate analog inhibitors often had reduced membrane permeability, poor chemical stability, and low affinity for the target enzyme. Moreover, the same donor is usually used by several glycosyltransferases, e.g., there are at least 16 different human *N*-acetylglucosaminyltransferases (GlcNAc-Ts, or GnTs) utilizing UDP-GlcNAc as the donor [398], which complicates the development of specific inhibitors based on the donor. Besides this, the synthesis of substrate analogs is usually a multi-step process, which further complicates their practical use.

Glycosyltransferases involved in the biosynthesis of minimal carbohydrate determinants sLe^x^ or sLe^a^ utilize UDPGalNAc, UDP-GlcNAc, UDP-Gal, GDP-Fuc, and CMP-NeuAc as donors. Various strategies that include structural modification of the transferred monosaccharide residue, the replacement of a phosphate unit, and altering the nucleotide residue were used to generate their mimetics. In alterations of a donor, the monosaccharide ring oxygen or glycosidic oxygen atoms were replaced by carbon to form carba-analogs of sugar-nucleotides. Another strategy was to replace monosaccharide hydroxyl groups with various substituents, e.g., a methyl group. A vast range of compounds was synthesized as potential inhibitors against GTs [389,391,392,393,399] but only a few promising results have been obtained. Most of these compounds exhibited *K*_i_ values similar to the *K*_M_ values of natural substrates in the micromolar range, and therefore, are not suitable as inhibitors. 

**Figure 16:**
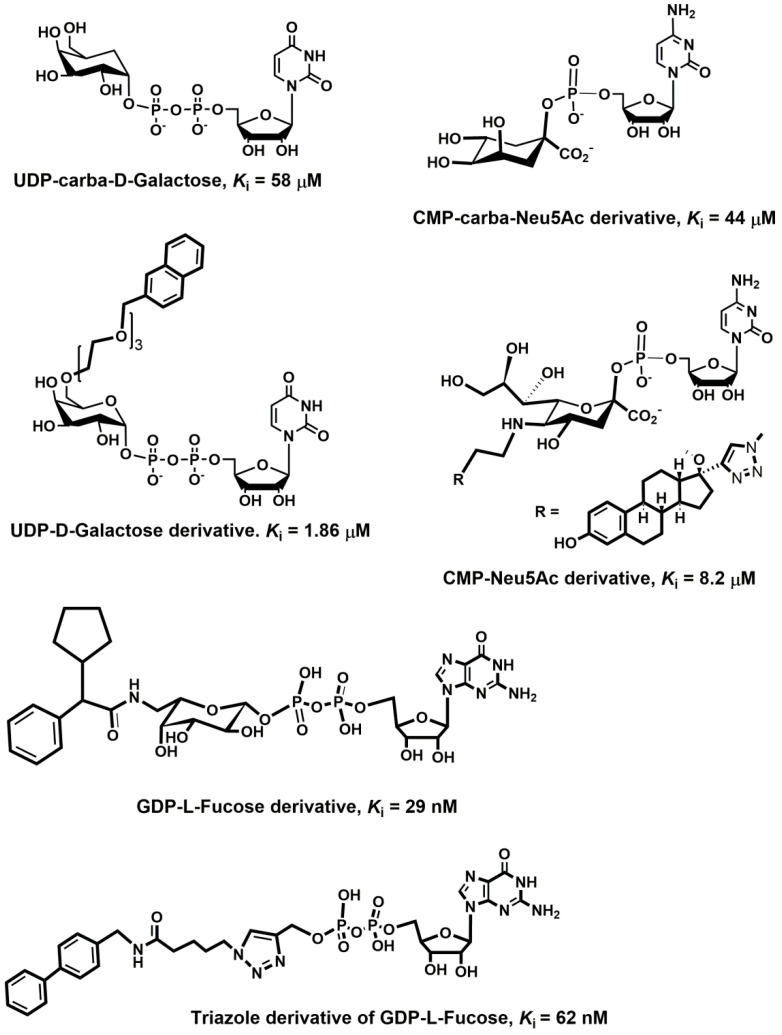
Schematic representation of selected donor-based inhibitors with modified monosaccharide residue UDP-carba-Gal [407], UDP-Gal derivative [408], CMP-Carba-Neu5Ac [409], CMP-Neu5Ac [410], Triazol derivative of GDP-Fuc [400], and GDP-Fuc [401].

Selected examples of synthesized compounds are given in Figure 16. The determined crystal structure of the *Helicobacter pylori* α-1,3-FucT [150] was used as a guide to developing FucT inhibitors [400]. The most potent inhibitors exhibited *K*_i_ = 62 nM and *K*_i_ = 29 nM against FucT-VI (Figure 16). Click chemistry was used to synthesize a FucT-VI inhibitor, a trialoze derivative with *K*_i_ = 62 nM [401].

**Figure 17:**
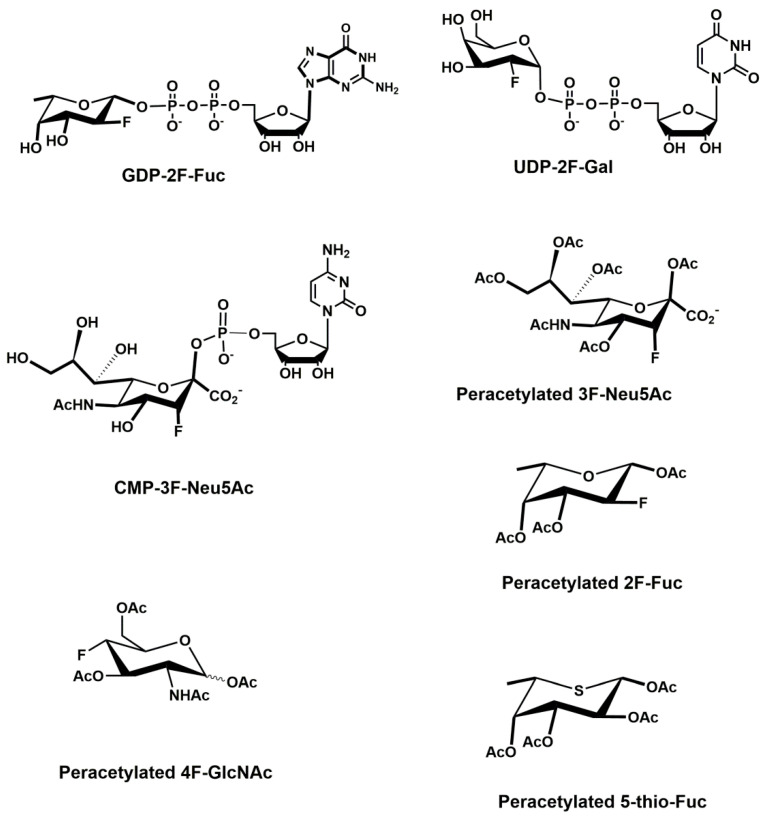
Schematic representation of fluorinated derivatives of donor-based inhibitors.

Analogous to glycosidases, where the glycosyl fluoride successfully inhibited their enzymatic action [402], fluoro derivatives of sugar-nucleotides were prepared (Figure 17). The synthesized UDP-[2F]-Gal, GDP-[2F]-Fuc, and CMP-[3F_ax_]-Neu5Ac exhibited competitive inhibition of β4Gal-T1, FucT, and ST6Gal, with *K*_i_ values in the micromolar range [403]. Peracetylated derivatives peracetylated-[2F]-Fuc, and peracetylated-[3F_ax_]-Neu5Ac were used as cell-permeable metabolic inhibitors of fucosyl- and sialyltransferase [404]. These compounds are intracellularly converted to the corresponding donor substrates GDP-[2F]-Fuc, and CMP-[3F_ax_]-Neu5Ac. In vitro experiments have shown that fluorinated analogs of fucose and sialic acid inhibited fucosyltransferases and sialyltransferase and thus alter the glycosylation patterns in the investigated cells. Also, the in vivo administration of 3F-Neu5Ac to mice decreases sialylated glycans in cells [405]. As a result, cells lost their selectin binding affinity, and leukocyte rolling was damaged. It has been recently shown that 5-carbamate derivatives of peracetylated-[3F_ax_]-Neu5Ac exhibited a prolonged and increased inhibition of STs in several cell lines [406].

Two monosaccharide derivatives [411,412] have been developed to inhibit the biosynthesis of sLe^x^ (Figure 17). One is a fluorinated analog of *N*-acetylglucosamine, peracetylated-4-fluorinated d-glucosamine (4F-GlcNAc). It has been shown that 4F-GlcNAc incorporates into the poly-*N*-acetyllactosamine chain and thus interferes with GlcNAc in the biosynthesis of terminal sLe^x^. The replacement of the O4 hydroxyl group with a fluorine atom in GlcNAc blocks the next addition of Gal to the C4 carbon atom of GlcNAc, leading to the termination of the sLe^x^ biosynthesis. It has been shown that 4F-GlcNAc reduces expression of the E-selectin ligand with high efficacy and prevents contact hypersensitivity in mice [412]. Another example, peracetylated 5-thiofucose (5T-Fuc), utilizes a different mode of inhibitory mechanism [413]. The authors showed that cancer cells utilize 5T-Fuc for the biosynthesis of GDP-5T-Fuc, which is an analog of the natural sugar nucleotide GDP-Fuc. While GDP-Fuc is the donor for the enzymatic reaction catalyzed by FucT, its 5-thio analog does not function as the donor. In contrast, GDP-5T-Fuc inhibited the transfer reaction at a low micromolar value that led to a decreased level of cell surface sLe^x^.

The contribution of electrostatic interactions between the diphosphate moiety and the metal cofactor or positively charged amino acids located in the catalytic site is crucial for the binding affinity of the donor. Various strategies were used to replace phosphate groups [389,393,414] with a phosphonate group, or a neutral group such as a monosaccharide, peptide bond, malonate or tartrate. Although these derivatives are more stable and their synthesis is reasonable, their inhibition potency was weak.

**Figure 18:**
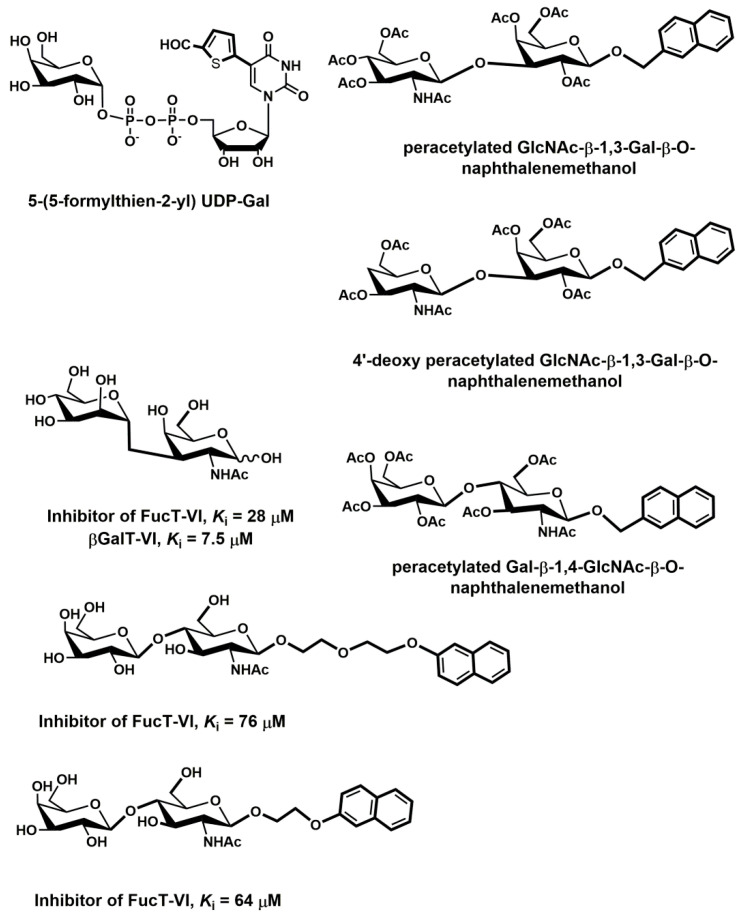
Schematic representation of substrate-based GT inhibitors.

The nucleoside base is crucial for the recognition of the donor by GT, and modifications of the base usually result in a loss of substrate activity. In general, the binding pattern means that there is only one position in the base that is suitable for alteration, e.g., in UDP, it is position C5 [415]. Therefore, inhibitors based on the variation of nucleobases are scarce. Recently, a structure-based design led to the discovery of a new model of GT donor-based inhibitors with *K*_i_ values in the low micromolar to nanomolar range [416,417,418,419]. These inhibitors (Figure 18) are characterized by a modification of the C-5 position at the uracil base. The crystallographic studies of β4-Gal-T suggested that glycosyltransferases with a GT-A fold, after binding the donor, often undergo a conformational change, in which an internal loop is structured and create the binding site of the acceptor [143,420]. The 5-substituted derivatives of uracil are intended to interfere with this movement and thus inhibit the enzyme. Although the potency of these inhibitors is not high, it was shown [421] that the β4-Gal-T inhibitor, the 5-substituted UDP-Gal analog 5-(5-formylthien-2-yl) UDP-Gal, attenuates PSGL-1 expression in human monocytes.

Oligosaccharides that function as acceptors for GTs involved in the synthesis of sLe^x^ have lower affinity than donor substrates. The corresponding *K*_M_ values are usually in the millimolar range. Several different approaches have been developed for creating acceptor-based inhibitors of GTs. The replacement of the nucleophilic hydroxyl groups participating in the enzymatic reaction is a natural choice for the development of inhibitors providing that the deoxygenated acceptors are recognized by GT. Several deoxygenated acceptor analogs were synthesized and their potency screened against eight GTs [422]. Surprisingly, only four GTs were inhibited and *K*_i_ values were similar to *K*_M_ values indicating weak inhibitory activity. The replacement of the proton from the anomeric hydroxyl group by hydrophobic aglycon seems to be the most successful strategy [423,424,425,426,427]. Peracetylated GlcNAc-β-1,3-Gal-β*-O-*naphthalenemethanol and peracetylated Gal-β-1,4-GlcNAc-β*-O-*naphthalenemethanol (Figure 18) inhibited β4Gal-T with *K*_i_ = 10 μM – 50 μM. It has been shown that these compounds are rapidly deacetylated by carboxyesterases [425,426] in cells. Deacetylated compounds then function as substrates for Gal-T and as a result, the micromolar concentration of these disaccharides reduces the expression of sLe^x^ using human adenocarcinoma cell lines. Also, these compounds attenuated the expression of sLe^x^ and, therefore, P-selectin-dependent cell adhesion in Lewis carcinoma cells [423]. It was observed that the 4-deoxy analog of GlcNAc-β-1,3-Gal-β*-O-*naphthalenemethanol binds to β4Gal-T [428]. Since in this compound the nucleophilic hydroxyl is missing, the catalytic reaction cannot proceed and the 4′-deoxy analog behaves as a competitive inhibitor. A series of peracetylated *N*-acetyllactosamine analogs with variation at the aglycon moiety weas synthesized to investigate their inhibitory activity against FucT [429]. Affinity measurements revealed that deacetylated analogs with naphthalene groups as the aglycon were the most potent inhibitors against FucT-VI. The naphthalene group linked with a three- and six-atom linker (Figure 18) exhibited *K*_M_ values of 64 mM and 70 mM, respectively.

Another approach, a so-called bisubstrate strategy, was designed to improve the specificity and potency of inhibitors [393,430]. In this concept, bisubstrate analog inhibitors consist of donor and acceptor mimetics that are covalently linked. The bisubstrate analog inhibitor is supposed to occupy the active site of the enzyme. Therefore, the three-dimensional shape of a bisubstrate analog should resemble the three-dimensional shape of the enzyme active site to allow the binding of bisubstrate analogs. Several substrate analog inhibitors were synthesized; however, assumptions of their high inhibitory activity prove to be incorrect. Their inhibition, with some exceptions, was weak, with *K*_i_ values in the millimolar range. Moreover, the synthesis of bisubstrate analogs usually requires a large number of steps and is relatively demanding.

**Figure 19:**
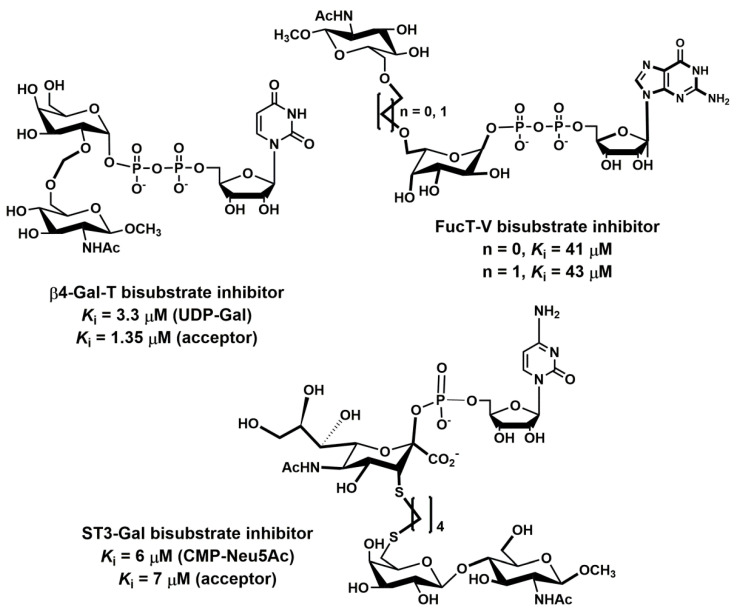
Schematic representation of selected bisubstrate analog inhibitors.

Linking together UDP-Gal and GlcNAc led to competitive bisubstrate analog inhibitors [431] (Figure 19) against β4-Gal-T with *K*_i_ values of 3.3 μM (UDP-Gal) and 1.35 μM (acceptor), respectively. In this inhibitor, the O-6 oxygen of the GlcNAc residue was connected to the C-2 hydroxyl group of the Gal residue via a methylene tether. Interestingly, when an ethylene tether was used, the inhibition was noncompetitive. A similar approach has been used for the synthesis of bisubstrate inhibitors against α1,3FucT [432]. In this case, the authors replaced fucose in GDP-Fuc with l-galactose, and the O6 oxygen of UDP-l-Gal and the O6 oxygen hydroxyethyl galactose were tethered with methylene or ethylene linker (Figure 19). The inhibitor with the methylene and ethylene linker against FucT-V was moderate, with *K*_i_ values of 41 μΜ and 43 μM, respectively. A series of bisubstrate analog inhibitors of sialyltransferases ST3 and ST6 were prepared by connecting the donor CMP-NeuAc and a disaccharide acceptor lactose and LacNAc [433]. The O-6 oxygen of the acceptor was linked to the O-3 oxygen of NeuAc through a sulfide bond separated by an alkyl linker with a variable length. The *K*_i_ values depended on the length of the linker and were in the range of 6–324 μM. Generally, *N*-acetylated analogs were more potent than lactose analogs. The analog with butylene linker (Figure 19) was a potent inhibitor of ST3 with the same *K*_i_ values of 6 μΜ for the donor (CMP-Neu5Ac) and 7 μM for the acceptor, respectively.

### 7.2. Non-Substrate Inhibitors

**Figure 20:**
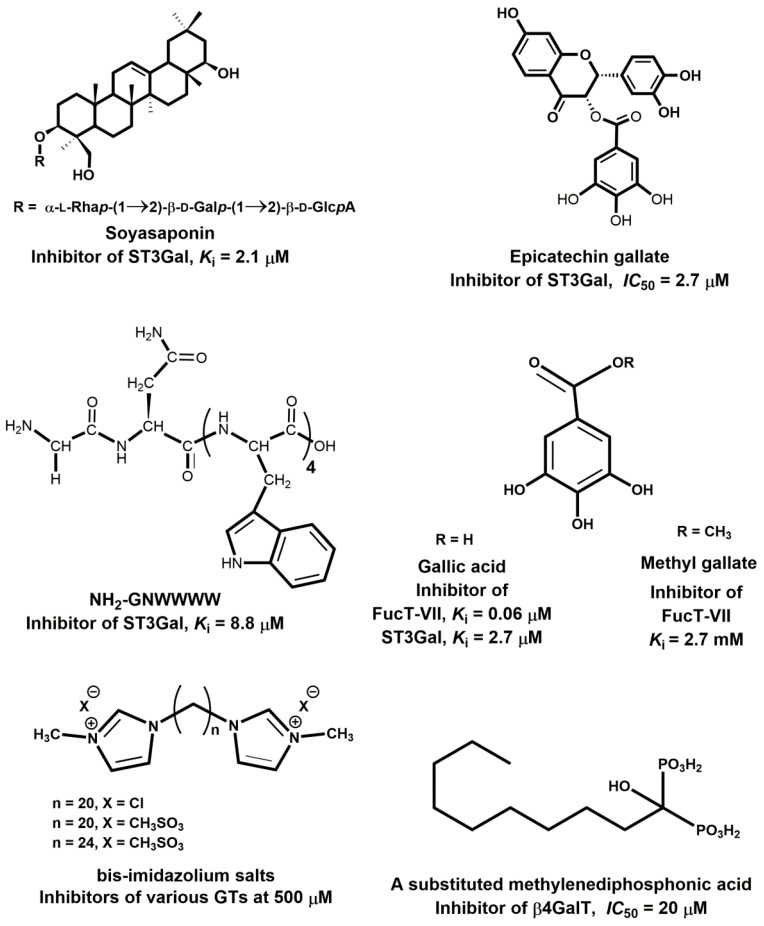
Schematic representation of selectin non-carbohydrate inhibitors.

Although plenty of GT inhibitors were developed as substrate analogs, their development into potential drugs has met with limited success. These compounds usually do not exhibit drug-like properties. Moreover, due to a lack of cell membrane penetration, they have a problem reaching the Golgi apparatus where GTs function. Therefore, attention was recently also focused on inhibitors that are not based on GT substrates [390,394,395]. However, although various chemotypes have been used, to date inhibitors have been developed only for a small number of GTs. For GTs involved in the biosynthesis of selectin carbohydrate determinants, mainly inhibitors of FucT and ST3 have been reported. Selected examples are given in Figure 20.

The screening of natural products led to several compounds that exhibited a low micromolar inhibitory activity against ST3. Soyasaponin I, from soybean, inhibits ST3Gal with *K*_i_ = 2.1 μΜ [434] and also specifically inhibited the transfer of sialic acid on murine melanoma cells [435] Soyasaponin I is a triterpenoid saponin with an α-l-Rha*p*-(1→2)-β-d-Gal*p*-(1→2)-β-d-Glc*p*A moiety attached at the 3-position of a pentacyclic system via a glycosidic linkage (Figure 20). Another steroid-based inhibitor, a derivative of lithocholic acid showed the noncompetitive inhibition of ST3Gal with *K*_i_ = 0.88 μΜ of metastasis cancer cells [436,437]. Different groups of ST inhibitors are gallic acid derivatives (Figure 20) that also exhibited inhibitory activity against ST3; e.g., a value of *IC*_50_ = 2.7 μΜ was measured for the flavonoid epicatechin gallate [438]. The hexapeptide NH_2_-GNWWWW (Figure 20) was identified by high-throughput screening with a *K*_i_ value of 8.8 μΜ and might represent the lead compound for the further development of potent and specific ST inhibitors [439].

It is noteworthy that gallic acid and several of its derivatives also inhibit FucT, e.g., gallic acid, methyl gallate, and epigallocatechin are inhibitors against FucTs and STs (Figure 20) with the *K*_i_ values in a low micromolar range [438]. Gallic acid and its derivatives are strong antioxidants with numerous biological activities [440] and this property might also be responsible for inhibition of some GTs. An interesting group of compounds, positively charged, bivalent imidazolium salts has been observed to be inhibitors of various GTs [441]. In these compounds, two imidazolium groups are linked with a long aliphatic chain. The three most potent compounds (Figure 20) strongly inhibited ppGalNAcT-1 and C2GnT-1, and moderately β4GalT-1 at a concentration of 500 μM. The high-throughput screening of a vast combinatorial library of compounds designed by rational design led to a new type of bisphosphonates as selective inhibitors (Figure 20) against β4GalT; (*IC*_50_ = 20 μM) [442]. Though there has been some progress in the development of non-substrate inhibitors for GTs, and new inhibitor chemotypes have been identified, this approach is still in its infancy. Before these inhibitors enter the drug refinement cycle, several vital questions must be answered. Probably the most relevant is to establish the selectivity of inhibitors against GTs and also against other enzymes. The mechanism of action is another issue that must be resolved. Since the activity of the vast majority of inhibitors was tested using recombinant enzymes, it is also crucial to evaluate their activity in cellular assays.

### 7.3. Transition State Analog Inhibitors

The best inhibitors of an enzymatic reaction are transition state (TS) analogs [443,444,445]. These molecules are expected to alter oligosaccharide structures by the efficient inhibition of enzymes involved in their biosynthesis. Therefore, a great deal of effort has been focused on the development of mimetics such as transition state analogs for enzyme inhibition [446,447]. The design of transition state analogs requires knowledge of the transition state structure of a given enzymatic reaction. It is difficult to determine the transition state structure; therefore, molecular modeling methods are used to provide information about the enzymatic mechanism at the atomic level [59,448,449]. The predicted structures of transition state models can be used as a guide for developing mimetics of the TS, transition state analog inhibitors (TSAIs).

**Figure 21:**
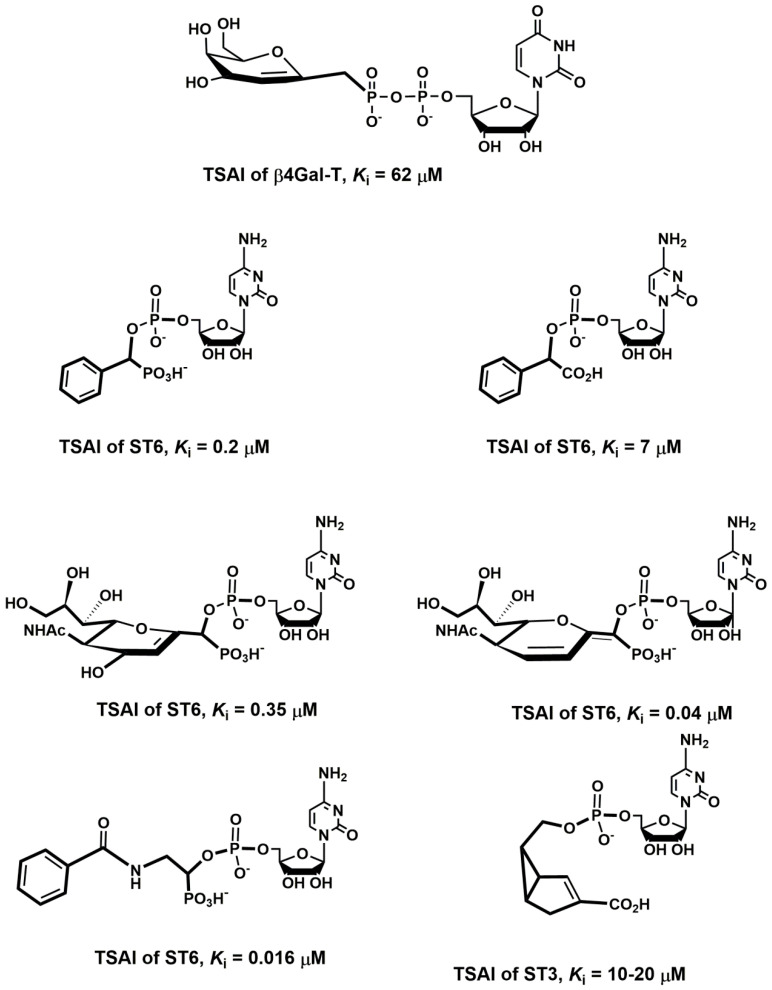
Schematic representation of selected transition state analog inhibitors.

**Figure 22:**
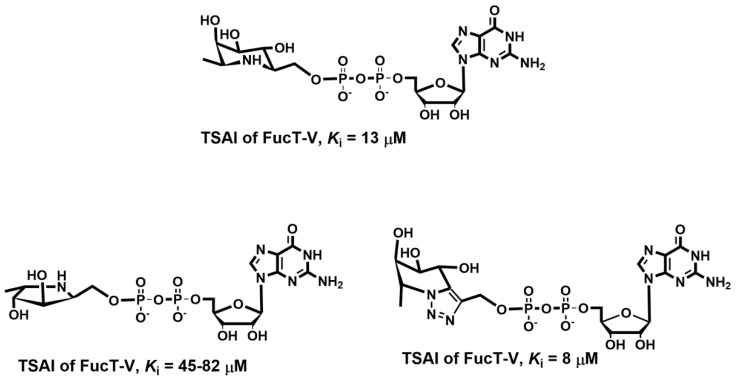
Schematic representation of selected transition state analog inhibitors.

A lack of transition state structures has hindered the development of transition state analog inhibitors, and only a few have been reported with *K*_i_ values. Until recently [60], transition state structures were not available for GTs involved in the synthesis of sLe^x^. Therefore, transition state analog inhibitors were prepared using suggested TS structures. The assumed TS characteristics were reflected in various approaches used to make TSAIs. These can be divided into four groups: TSAIs with the ring shape of the transferred saccharides distorted into a quasi-planar conformation with an *sp*^2^ anomeric carbon; TSAIs with an elongated C1-O glycosidic bond; TSAIs with a positive charge on the C1 anomeric carbon; and TSAIs with phosphate moieties replaced by a functional moiety. Often TSAIs contained a combination of the above features. Selected examples of synthesized TSAIs for the relevant GTs with inhibitory activity in the low micromolar range will be discussed and are given in Figure 21 and Figure 22.

One of the first TSAIs was prepared against β4Gal-T (Figure 21) as the C-glycoside analog of UDP-Gal with the galactose residue replaced by a residue with a double bond to provide a planar structure in the transferred saccharide [450]. Several TSAIs against sialyltransferases were synthesized [451,452] with replacing the NeuAc residue in CMP-NeuAc with an aromatic ring. (Figure 21) Their *K*_i_ values against ST6 were in the range of 40–350 nM. A series of proposed TSAIs were synthesized where a simple amide mimicked an oxocarbenium ion in the TS [453]. The best compound (Figure 21) exhibited *K*_i_ = 0.016 μM, ~2600-fold higher affinity to ST6 than CMP-NeuAc, with a *K*_M_ value of ~41 μM. Unfortunately, the *K*_i_ values against ST3 were not reported. The elongation of the C1-O glycosidic bond by adding a single methylene group between the anomeric carbon and the phosphate oxygen gave TSAI against ST3 with *K*_i_ = 10–20 μM [454]. The same approach was used to prepare TSAIs against FucT [455]. The synthesized compounds were competitive inhibitor against FucT-V and FucT-VI with *K*_i_ between 8 and 13 μM (Figure 22).

The above-discussed compounds were designed to mimic one or more structural characteristics of the donor in the transition state of a particular glycosyltransferase. The expectation was that such compounds might be transition state analog inhibitors. However, the obtained low or moderate inhibitory activity suggests that these compounds do not represent true TSAI, and could be considered to be bisubstrate analogs. TSAIs should exhibit much stronger inhibitory potency, with a *K*_i_ value in the picomolar range or at least in the low nanomolar range.

The biosynthesis of selectin minimal recognition determinants, the tetrasaccharide sLe^x^ and its analogs requires the coordinated action of several glycosyltransferases (see paragraph 4), such as ppGalNAcT2, Core-1GalT, Core-2GnT, β4Gal-T1, FucTs, and GlcNAc6ST. Thus, these glycosyltransferases are targets for designing transition state analog inhibitors as potential therapeutics. The investigated catalytic mechanisms of several GTs and the determined transition state structures have been reviewed recently [59,60].

**Figure 23:**
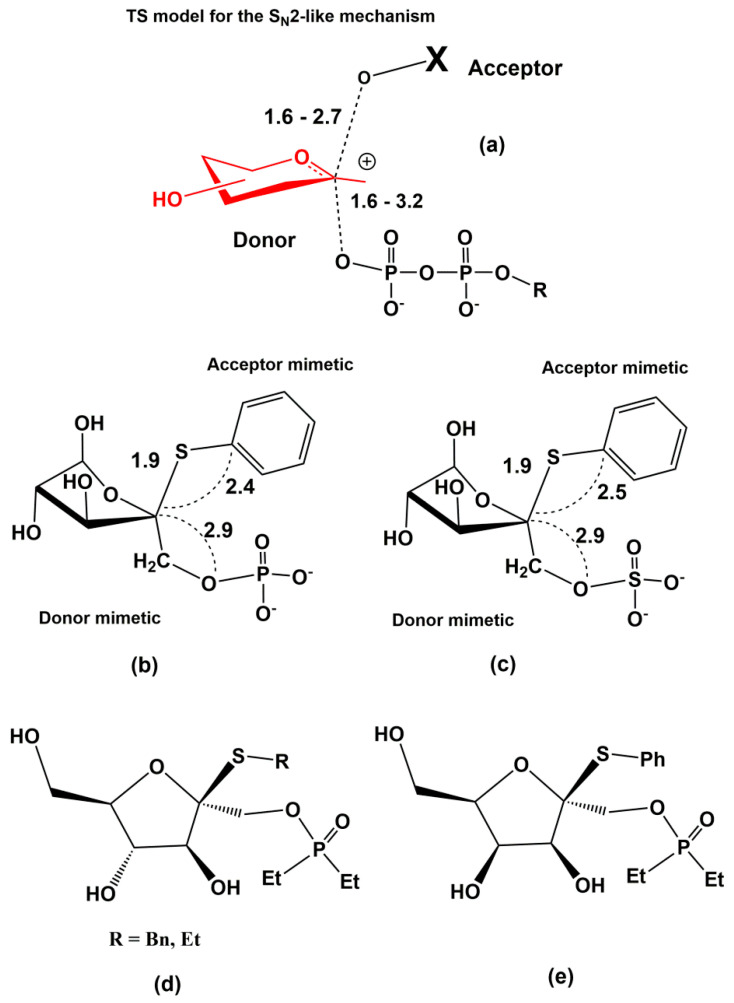
Schematic representation of (**a**) transition state structures for retaining GTs utilizing the S_N_2 reaction mechanism, (**b**,**c**) the proposed scaffolds of potential TSAIs, (**d**,**e**) potential transition state analog inhibitors of *N*-acetylglycosyltransferses. Numbers represent relevant distances in Å.

The results showed that a transition state model consists of both the donor and acceptor that have their structures distorted compared to the ground state structures and are linked together with non-equilibrium linkages in a specific orientation. Investigations of the catalytic mechanism of GTs provided the following general structural features of the TS models [59,60] (Figure 23a): (a) the transferred monosaccharide ring is flattened, and its conformation resembles a deformed chair/envelope conformation with oxo-carbenium character at the sp^2^ hybridized anomeric carbon; (b) the C1–O1 bond is elongated compared to the standard C–O bond length; (c) the formed glycosidic linkage is also longer than the standard bond length; (d) both, the forming and breaking bonds are oriented almost perpendicularly to the plane defined by the C2-C1-O5-C5 atoms.

Based on these structural characteristics, two transition state scaffold models have been proposed [456,457] (Figure 23b,c). The scaffold structures resemble structural features of TS: the distance C1-O1 is elongated by adding a methylene group, the distance between the anomeric carbon C1 and the acceptor oxygen is enlarged by replacing the oxygen with sulfur atom. Thus the distances around the anomeric center in the scaffolds are similar to those in TS models. A deformed six-membered hexopyranose ring was replaced with a five-membered furanose ring. Several potential inhibitors based on these scaffolds have been synthesized [458,459,460]. Two examples of potential inhibitors are given in Figure 23d,e. However, further studies are necessary to obtain the transition state analogs of the enzymes involved in the biosynthesis of sLe^x^ with the required potency, specificity, and drug-like properties.

## 8. Summary and Perspectives

The extraordinary efforts of many research groups over the last three decades have led to progress in our knowledge of selectins’ roles and clarified the structure, properties, behavior, and biological functions of selectins. Selectins are well-characterized adhesion molecules that mediate the interactions of leukocytes and cancer cells with a vascular wall in various physiological and pathological processes. The obtained results, based on multidisciplinary approaches that used structural, biochemical, enzymatic, genetic, and molecular modeling methods, clearly revealed that the selectins exhibit a dual role or Dr. Jekyll and Mr. Hyde behavior; they are required for a healing process (Dr. Jekyll), but also are involved in the development of severe diseases (Mr. Hyde). Though the adhesion of selectins in these processes is controlled by carbohydrate determinants, such as sLe^x^ and sLe^a^, a detailed structure of the physiological ligands and the regulatory processes of their biosynthesis have not been fully determined. The involvement of selectins as adhesion receptors that mediate inflammatory diseases and metastasis made the given selectin therapeutic targets for these diseases. The development of real inhibitors of selectin-ligand interactions in vivo is a challenging task due to the dual role of selectins. On the one side, extremely potent inhibitors might have undesired effects on healing processes. On the other hand, weak inhibitors might not intervene sufficiently in the pathological processes of severe diseases. Therefore, effective therapeutic agents may require well-balanced action against selectin-ligand interactions. Moreover, the results presented here imply that a clinically successful inhibitor has to inhibit at least two selectins. Also, the structural features of the calcium-dependent carbohydrate recognition domain that has a shallow surface make the rational design of selectin inhibitors difficult. A plethora of diverse glycomimetic or non-carbohydrate, and polysaccharide inhibitors that inhibit selectin-ligand interactions have been developed and despite this considerable effort only a few compounds have showed promising results in clinical trials. Recent progress in the understanding the catalytic mechanism and determining the transition state structure of the enzymatic reactions involved in the biosynthesis of carbohydrate determinants of selectin counter-receptors offers potential approaches to the therapeutic intervention of inflammatory diseases and tumor progression and metastasis. A combination of glycobiology, medicinal chemistry, and molecular modeling will provide a guide for the rational design of such agents.
